# Religious and spiritual beliefs and attitudes towards addiction and addiction treatment: A scoping review

**DOI:** 10.1016/j.abrep.2021.100393

**Published:** 2021-11-14

**Authors:** Jennifer T. Grant Weinandy, Joshua B. Grubbs

**Affiliations:** Bowling Green State University, USA

**Keywords:** r/s, religious and spiritual, Religion and spirituality, Addiction, Addiction treatment, Attitudes

## Abstract

**Introduction:**

Throughout history, ideas about addiction have been informed and influenced by religious belief and practice. Even continuing into the present, religion and spirituality are often thought to impact attitudes towards addiction and its treatment, particularly given the use of religion and spirituality in various well-known treatments such as Alcoholics Anonymous. Although research has flourished with regards to the role that addiction and spirituality might play in vulnerability to addiction or the treatment of addiction, there has been comparatively less research examining how religion might impact attitudes toward addiction more broadly.

**Method:**

The present work sought to examine the current state of empirical literature evaluating the relationships between religion and attitudes toward addiction and addiction treatment. In service of the above aim, a scoping review was conducted.

**Results:**

This summary of peer-reviewed, quantitative studies (*n* = 36) found that higher religiosity is related to more belief in the disease model of addiction in providers, negative attitudes towards addiction, and a stronger support for spiritually-based treatments. However, results varied based on the measures used and many relationships tested were not significant. There also appeared to be differences in the interaction between religion and spirituality and other variables, such as the respondent’s sex or whether the individual was a student or provider, in terms of attitudes.

**Conclusions:**

Further research is needed to fully understand the nuances in the relationship between these variables, including more clear operationalization and standardized measurement. Until such research is conducted, no cohesive conclusions can be drawn, and clinical implications remain unclear.

## Highlights

• A large proportion of addiction treatment in the U.S. is at least somewhat religious in nature.
• Some evidence suggests that attitudes toward addiction and addiction treatment are influenced by religious beliefs.
• Relatively little prior research has examined how religion may shape attitudes toward addiction.
• Some research suggests that religious beliefs are associated with negative attitudes toward addiction.
• Future research is urgently needed to understand how religious beliefs may influence attitudes toward addiction treatment.

## 1 Introduction

In 2017, roughly 6% (19.7 million people) of Americans over the age of 12 met criteria for a substance use disorder in the previous 12 months (Substance Abuse and Mental Health Services Administration, 2018). Similar research with behavioural addictions^1^ suggests approximate population prevalence rates in the United States of 1–3% for Gambling Disorder (Welte, Barnes, Tidwell, Hoffman, & Wieczorek, 2015), 0.3–1% for Internet Gaming Disorder (Przybylski, Weinstein, & Murayama, 2017), and 3–6% for Compulsive Sexual Behaviour Disorder (Kuzma & Black, 2008). Each of these figures represent millions of individual people within the United States alone, and similarly high numbers found throughout the world suggest that addictions are a significant contributor to global disease burden (Degenhardt et al., 2018). Ultimately, recent estimates suggest that addiction in the United States bears an economic cost of over a trillion dollars a year in medical expenses, crime, lost productivity, and loss of life (Recovery Centers of America, 2016). Given the frequency of these disorders, their enduring presence in the public consciousness, and the costs associated with them, there is a persistent need for research into domains that may affect those suffering from addiction or provide relief and recovery for them. One such domain of interest is religion and spirituality (hereafter: religion/spirituality)^2^.

In recent years, there has been a notable increase in the study of religion and addiction (e.g., Benda and McGovern, 2006, Faigin et al., 2014, Grubbs et al., 2017, Stauner et al., 2019). Of note, this increase is in empirical attention, as various religious and spiritual (r/s) communities around the world have recognized for millennia that behaviours and substances may be potentially addictive and cause negative consequences (e.g., Sura Ma’idah 5:90–91). Throughout history, r/s beliefs and attitudes have affected the way many people see the world and view others’ behaviours, particularly with regards to addiction. For example, the American temperance and prohibition movement is often viewed to have stemmed from religious, Protestant beliefs (Schmidt, 1995); and the most ardent believers in the potentially addictive nature of pornography or sexual behaviours tend to be the highly religious (e.g., Bradley, Grubbs, Uzdavines, Exline, & Pargament, 2016). However, between and within religions, there is variability surrounding which substances and behaviours should be avoided or followers should be wary of, with some faith traditions having codified, explicit instructions about what followers should do with those who are addicted (Royce, 1985).

Importantly, the effects of religion on addiction treatment are likely amplified in the United States, relative to many other affluent Western nations, as many of the largest substance abuse and behavioural addiction treatment and recovery programs are affiliated with religion either formally or informally. For example, the r/s influences in 12-step programs, such as AA and GA, are well-documented (e.g. Dossett, 2013, Witkiewitz et al., 2016), and promotional materials for Celebrate Recovery (an evangelical Christian based 12-step addiction recovery program) boast a membership of 29,000 churches in the United States and a participant reach of over 3.5 million people (www.celebraterecovery.com). Furthermore, research suggests that aspects of spirituality are useful and encouraged within addiction treatment (e.g. Heinz et al., 2010). Additionally, r/s traditions likely influence the addiction attitudes of more secular individuals. For example, a strong, overt r/s tradition’s presence in a society may impact political policies and, therefore, the views of more secular individuals who live there. As such, r/s beliefs and attitudes likely influence how individuals and societies view, and treat, those with addictions, regardless of the individual attitudes of the persons with addiction or the persons treating those with addictions. Based on the above factors then, there is a need to understand how religion/spirituality affects individuals’ views of addiction and how it should be treated. Based on this need, the purpose of the present work is to conduct a scoping review of current literature that examines how individual r/s beliefs influence attitudes toward and beliefs about addiction, both in the United States and other countries.

### 1.1 Religious and spiritual beliefs and attitudes towards addiction

There are references to addiction in five of the major world religions, as well as references to when substances or behaviours are encouraged or should be used with caution. There are, however, differences in views and strengths of attitudes surrounding what could become addictive or is helpful in r/s traditions. For example, some traditions suggest that behaviours and substances are acceptable if they are done in moderation and some even use substances or behaviours to access their Higher Power or create community and raise money. In some Native American spiritual traditions, some psychoactive substances may be used in some circumstances to experience a better connection to Earth, the self, or others, and thus bring understanding (Oklevueha Native American Church Sacrament, n.d.). Similarly, Judeo-Christian traditions often use alcohol in rituals, such as the Jewish Passover festival and the celebration of the Eucharist in Catholic churches. In contrast, some Christian denominations disagree with the use of alcohol during any sacraments and suggest that such practices may lead to a problematic relationship with alcohol and therefore instruct followers to refrain from drinking (e.g., United Methodist Church). Others still are silent on the matter and leave it to the follower’s conscience to decide whether to use alcohol (e.g., British Baptist Union).

Similar patterns as above are evident in behavioural addictions as well. For example, gambling is sometimes accepted as a form of raising money in Christian traditions, whereas it is not acceptable in others (Cohen & Schwartz, 2018). Similarly, religious groups often encourage specific forms of sexual behaviour. For example, in some Buddhist traditions, tantra is used as a way of having sex to attain enlightenment (Douglas & Slinger, 1979). Many other r/s traditions encourage individuals to have sex within the context of heterosexual marriage to encourage a healthier relationship, both between the people involved and with the Higher Power, as well as to avoid possibly harmful sexual behaviours (Pope John Paul II & Waldstein, 2006). Yet, many other forms of sexual behaviours (i.e., pornography use) are explicitly condemned, with the addictive potentials of such behaviours often being invoked as a reason for religious opposition to their use. Further complicating matters, many r/s traditions are silent on the majority of specific behaviours that may be considered addictive, such as video game play (Grubbs & Grant, 2020). In sum, though many belief systems across the globe often acknowledge addiction and, in many cases, have strong proscriptions around engaging in addictive behaviours or processes, there is little consistency across or within religious traditions regarding the pantheon of potentially addictive behaviours. As such, it is difficult to draw clear inferences regarding the effects of religious belief on perceptions of addiction and addiction treatment.

### 1.2 Religious and spiritual views of addiction treatment

There are several theoretical models that influence how the scientific and medical communities view addiction and addiction treatment. These most commonly include:1) the Medical/Disease model, which suggests that the substance or behaviour effects the brain’s structure or functioning (e.g., Volkow & Morales, 2015) directing practitioners towards pharmacology treatments (e.g., Grant et al., 2014, Jorenby et al., 1999); 2) the Moral/Ethical model, which focuses on whether an action is morally “good” or “bad,” and views addiction as a sin or the behaviour/substance as an evil, leading to various treatment suggestions, which in the more religiously based versions tends to be based on religious teachings (e.g., Iran Human Rights Documentation Center, 2012, The United Methodist Church., 2016); and 3) the Biopsychosocial model, which emphasizes the role of social and psychological factors as well as brain functioning (e.g., Blaszczynski & Nower, 2002) supporting the use of therapy alongside pharmacology (e.g. Carroll et al., 2003) and the importance of social connections, such as AA (Tracy & Wallace, 2016). Not surprisingly, it seems that religion might influence which of these models any given individual places credence in.

R/s views often influence notions of how to treat addiction or those thought to be acting in a way that could become addictive. Oftentimes this mirrors the r/s tradition’s beliefs and attitudes towards the behaviour or substance itself. That is, oftentimes a religious group’s teachings about substance use informs how that group approaches the treatment of individuals addicted to such a substance. For a particularly extreme example, Articles 136 and 264 of Iran’s Penal Code suggest, by invoking Islamic Sharia law, that a person convicted of drinking three times must receive the death penalty (Iran Human Rights Documentation Center, 2012). In a more general example, AA (which is deeply religious in many regards, e.g., Dossett, 2013, Witkiewitz et al., 2016) notes that it works towards abstinence only for the sake of restoring a healthy relationship with the Higher Power (Alcoholics Anonymous World Services Inc, 2018). Moreover, as noted above, r/s traditions administer and manage many treatment facilities and recovery programs (e.g. AA; Timberline Knolls, 2019). These programs often offer a range of resources, from support groups to medication-assisted recovery programs. Additionally, some r/s traditions are moving towards an acceptance of harm reduction and maintenance practices. For example, some mosques are now being used as centres for medication-assisted recovery, including methadone maintenance (Rashid et al., 2014).

R/s traditions often manage behavioural addiction treatment centres and programs. For example, Lutheran Social Services manages one of the largest gambling counselling networks in North Dakota (www.gamblernd.com) and Pure Life Ministries works with individuals to reduce sex addiction symptoms (www.purelifemisitries.org). Similarly, in an extreme example, Kyle Long (the alleged shooter in several Atlanta area spas in March of 2021) spent substantial time in an evangelical, inpatient sex addiction treatment program in the greater Atlanta area (Berman, Shammas, Armus, & Fisher, 2021). Importantly, however, the behaviour that is the focus of an addiction may strongly influence the type of treatments viewed as acceptable by the religious group involved in treatment. The United Methodist Church’s Social Principles states that “As an act of faith and concern, Christians should abstain from gambling and should strive to minister to those victimized by the practice.” Therefore, even though they teach against gambling at any point, they also recognize the difficulty of addiction and encourage their followers to help gamblers to attain abstinence. Conversely, in the case of sexual addiction, it is more likely that r/s traditions would support moderate use, within a church sanctioned relationship, given that the behaviour is more socially accepted and seen as central to human nature (e.g. Pope John Paul II & Waldstein, 2006).

### 1.3 The present work

Given the above literature, it is reasonable to conclude that religion/spirituality influence attitudes toward addiction and its treatment. As such, the primary purpose of this work was to systematically review available literature and synthesize such literature into clear findings related to the multifaceted relationships between attitudes towards addiction and individual religion/spirituality.

At present, some literature reviews exist discussing research about religion, spirituality, addiction, and addiction treatment more generally. These have looked at potential r/s protection or support for those using substances (Seva Diaz & Vazquez Casabona, 1975), participating in certain addictive behaviours (Karaga, Davis, Choe, & Hook, 2016), and those with an addiction (McBride et al., 1996, Unterrainer et al., 2013). Other reviews have focused on how religion/spirituality act upon those attending r/s treatment programs such as AA (Feigenbaum, 2013) or the availability of various treatment modalities (Alam-mehrjerdi, Noori, & Dolan, 2016). Still others focus on how religion/spirituality may affect mental health or behaviours more generally, focusing on their protective potential (AbdAleati et al., 2016, Koenig, 2001). More historical perspectives have also been taken, often looking at socio-political perspectives of specific substances or behaviours in a certain geographic area (Baumeister and Placidi, 1983, Heath, 1974, Pontell and Geis, 2008).

A study aimed at compiling a bibliography of literature about addictions, religion, and spirituality found 101 articles which discussed attitudes towards substance use, spirituality, and religion (Geppert, Bogenschutz, & Miller, 2007). This study suggested that much of the literature pointed to negative attitudes towards substance use, particularly by certain r/s traditions. Another study did complete a systematic review, but they focused on youth attitudes and beliefs about substance use in the Eastern Mediterranean region (El Khoury, Noufi, Ahmad, Akl, & El Hayek, 2019). The authors found five eligible studies looking at general attitudes and beliefs rather than r/s attitudes and beliefs alone.

These reviews suggest that the domain of religion/spirituality is important when looking at attitudes and beliefs about addictions and addiction treatment. Specifically, a focus on attitudes towards addiction, rather than use of addictive substances or behaviours, is important given that treatment of those with an addiction may be dictated by r/s beliefs and such influences must be better understood to aid in guiding appropriate ways to incorporate religion/spirituality into treatment and policy decisions. However, no broad scoping review has been completed on r/s attitudes and attitudes towards addictions and addiction treatment. As such, this study aims to synthesize current research on how r/s beliefs and attitudes affect religious individuals’ view of addiction and addiction treatment. In particular, it aims to better understand potential differences between attitudes towards substances and behaviours of those in specific populations (i.e., providers, those seeking treatments, students, etc.), of those who identify with different religious affiliations, and between countries.

## 2 Method

In service of the above goals, we conducted a scoping review based on the PRISMA guidelines (Moher et al., 2009, Page et al., 2020). We used the search criteria from El Khoury, et al.’s (2019) study to inform the search criteria for this present effort, along with other keywords from known relevant studies (e.g. Bilal, Makhawi, Al-Fayez, & Shaltout, 1990).

### 2.1 Planned procedure

**Fig. 1:**
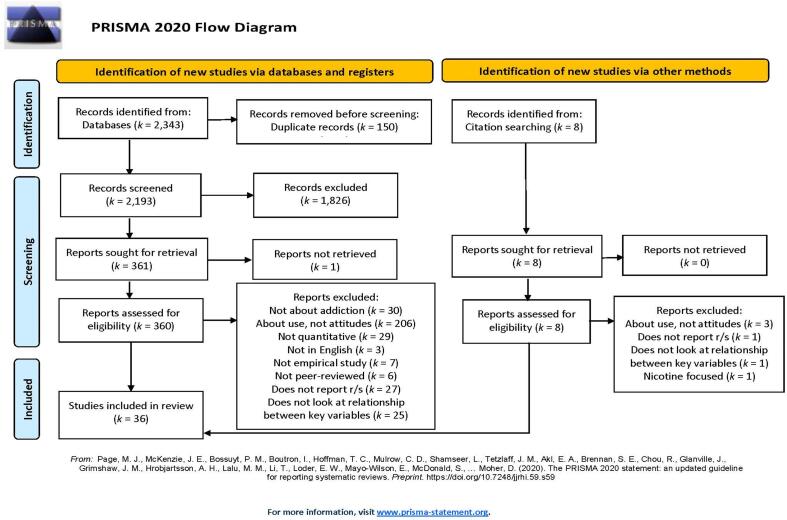
PRISMA 2020 Flow Diagram detailing search process for this literature review.

We conducted a literature search on January 1st, 2021, using PsycINFO, CINAHL, and PubMed, which found 2,343 studies (see Fig. 1 for PRISMA flowchart). These databases were searched using at least one term from each of the following: 1) “addiction,” “treatment,” “substance use disorder,” “drug,” “alcohol,” “misuse,” “abuse,” “dependence,” “gambling,” “gambling disorder,” “gaming,” “gaming disorder,” “sexual,” “compulsive sexual behaviour^3^ disorder,” and “behavioural addiction;” 2) “religious”, “religion,” and “spiritual;” and 3) “perceptions,” “acceptance,” “acceptability,” “stigma,” “conviction,” “view,” “opinion,” “belief,” and “attitude.” After removing duplicates, the articles’ titles and abstracts (*n* = 2,193) were screened for relevancy to this study, using the following inclusion criteria. All included studies were required to be empirical, include quantitative results for r/s beliefs and attitudes, available in English, discuss attitudes or beliefs towards an addiction or addiction treatment (i.e., as opposed to use alone), report a r/s variable, and be peer-reviewed. We excluded case reports, case studies, non-peer-reviewed reports (i.e., reports from commissions or agencies published as white papers or dissertations), reviews, conference presentations, book chapters, books in general, empirical articles that solely focused on an individual’s behaviour with regards to addiction (rather than attitudes/beliefs), or empirical articles that only focused on nicotine addiction^4^. Following this, the full texts of the articles (*n* = 367) were reviewed using the same eligibility criteria. Then the references of relevant papers were reviewed for other potentially relevant articles using the above steps (identified for further screening: *n* = 8; retained: *n* = 2). Screening and review of the articles were completed by one of four reviewers (i.e., the articles were split between reviewers), who then consulted with the first author to ensure continuity and make final decisions regarding inclusion. Finally, the eligible studies (*n* = 36) were summarized and synthesized by the first author.

## 3 Results

**Fig. 2:**
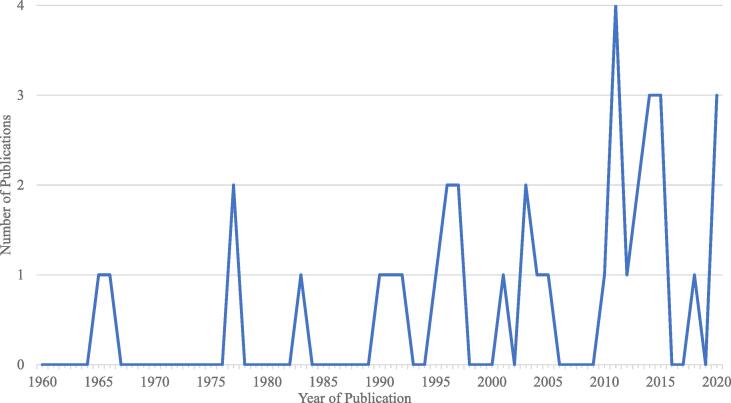
The number of publications found published in each year looking at the intersection between religion and spirituality and addiction and addiction treatment.

Included studies’ methods, sample characteristics, study design, and main findings pertaining to religion/spirituality and attitudes towards addiction are available in Supplementary Table 1. As suggested by Fig. 2, there has been varying degrees of interest in the intersection between attitudes towards addiction and religion/spirituality. Overall, 2 studies looked at behavioural addictions, 31 looked at substance addictions, and 2 looked at both behaviours and substances in terms of attitudes. Most reported attitudes towards alcohol (*n* = 24), followed by drugs or substance use generally (*n* = 12), cannabis (*n* = 4), pornography or sex (*n* = 3), gambling (*n* = 2), cocaine (*n* = 2), and the following were the focus of only one study: heroin, narcotics, the internet, video games, shopping, and food. Notably, one study looked at addiction in general, without specifying a substance or behaviour (Diallo, 2013), two studies included smoking/nicotine in their definition of general addiction (Bilal et al., 1990, Russell et al., 2011), and some studies looked at attitudes towards multiple substances or behaviours, usually to measure general attitudes towards addiction (*n* = 9). Further, 25 studies assessed the relationship between religion/spirituality and attitudes towards addiction itself, 27 studies assessed religion/spirituality’s relationship with attitudes towards addiction treatments, and 16 studies assessed both.

### 3.1 Demographics

Most of the studies were conducted in the USA (*n* = 22), followed by the United Kingdom (*n* = 5), Australia (*n* = 3), Israel (*n* = 2), Brazil (*n* = 2), Canada (*n* = 2), and only one study was conducted in the following countries: Kuwait, Russia, Norway, and India. Two samples were taken from multiple countries (Russell et al., 2011, Schaler, 1996, Schaler, 1997). Further, one study was conducted on several United States military bases in Europe (Globetti, Alsikafi, & Christy, 1977).

Overall, participants (Total *N* = 14,894) generally ranged in age from 20 to 50 years old, except in one adolescent sample (Globetti et al., 1977). Notably, two studies (Schaler, 1996, Schaler, 1997) appeared to use the same sample. Approximately half the participants identified as male; however, 5 studies did not report participant gender, and no studies reported including any participants who identified by a non-binary gender. The majority of the studies did not report participant sexual orientation; however, one study did include this demographic and the majority of participants identified as heterosexual (Droubay & Butters, 2020). The majority of participants self-identified as Caucasian or White, ranging from 48.8% (Lucchetti, Koenig, Pinsky, Laranjeira, & Vallada, 2014) to 94.9% (Schaler, 1996, Schaler, 1997). Nevertheless, seven studies (Dermatis et al., 2004, Dermatis et al., 2010, Flórez et al., 2015, Goldfarb et al., 1996, Lucchetti et al., 2014) reported that the majority of participants were not White. One study from Israel (Edelstein et al., 2020) reported participant identification as Jewish or Arab and another study from Kuwait (Bilal et al., 1990) reported participant identification as Arab or Kuwaiti. Finally, 17 studies did not report race or ethnicity.

Most of the studies included Christian individuals (*n* = 23), with seven studies utilizing Christian-only samples (Catholic only: *n* = 2; Protestant only: *n* = 1). Of these studies, 16 specified the inclusion of Catholic individuals, 14 specified Protestant individuals, and each of the following denominations were specified in at least one of the studies: Latter Day Saints, Mormon, Evangelical, Non-Evangelical, Lutheran, Methodist, Disciples of Christ, Episcopalian, Presbyterian, Congregationalist, Holiness, Pentecostal, and Adventist. Twenty-one studies also included non-Christian samples: Muslim (*n* = 10; Muslim only: *n* = 1), Jewish (*n* = 11), Hindu (*n* = 2), Buddhist (*n* = 1), Agnostic (*n* = 4), Atheist (*n* = 4), no affiliation (n = 9), and “other” (*n* = 12). Finally, ten studies did not report the religious affiliation of their sample.

In terms of populations, 6 studies sampled individuals seeking treatment for an addiction disorder, 18 sampled treatment providers, 9 sampled university/college students, 1 sampled adolescents who were in school, 7 sampled specifically religious populations (e.g., priests), and 5 sampled from the general population or other niche populations, such as teachers or specific racial groups.

### 3.2 Methodology of included studies

A sizable number of studies did not report key values, such as means or percentages, for key variables (*n* = 7). Most studies reported religious affiliation (*n* = 26), of which 18 studies used this as one of their religion/spirituality variables in the analyses. Other than using religious affiliation, studies predominantly used the Religious Belief Salience scale as a measure of religion/spirituality (*n* = 5), followed by the Spiritual Beliefs Scale (*n* = 3), the Adapted Spirituality Self-Rating Scale for Spiritual Orientation (*n* = 3), the Religious Background and Behaviour Scale (*n* = 2), Gallup Poll questions regarding religion/spirituality (*n* = 2), and then the following scales were each used once: the Spiritual Beliefs Questionnaire, the Santa Clara Strength of Religious Faith Questionnaire, the Beliefs and Values Scale, the adapted Religious Observance Scale, and the Religious subscale of the Arab-Muslim Attitudes Scale. However, other than those using affiliation only (*n* = 12), most studies used at least one non-validated measure or a single item question (*n* = 13), such as: importance of religion (*n* = 3), religious attendance (*n* = 7), r/s practices (*n* = 3), belief in a higher power (*n* = 3), r/s connectedness (*n* = 1), or self-perceived religiosity (*n* = 4).

In terms of measuring attitudes, ten studies utilized one of nine previously validated measures, including: the Marcus Alcoholism Questionnaire, the Seaman- Mannello Nurses’ Attitudes toward Alcoholism Scale, the Shortened version of the Alcohol and Alcohol Problems Perception Questionnaire, the Brazilian version of the Internalized Stigma of Mental Illness Scale adapted for Substance Dependents, the Modified Alcoholism Stigma Scale, the Community Behavioural Inventory, the Scale for Assessment of Attitudes toward Drinking and Alcoholism, the Addiction Belief Scale, and the RADAR research group’s Beliefs about Cannabis measure. A further four studies used an adapted version of five other measures (Attitudes towards treatment focused: *n* = 2) and five studies used three measures which were not validated but were used by authors previously (Attitudes towards treatment focused: *n* = 1). Three studies each attempted to validate a new measure of attitudes towards addiction as part of their paper. However, most studies (*n* = 20) again used a non-validated measure or a single item question (Attitudes towards addiction: *n* = 12; Attitudes towards treatment: *n* = 25). As such, overall, 57 different ways of measuring attitudes towards addiction were used, of which 28 focused on addiction treatment.

All studies employed cross-sectional designs; however, three studies used secondary data sets and three studies manipulated a variable (i.e., presenting different vignettes). Nine studies did not report at least some of their analyses’ results in full, four of which only omitted non-significant values. Many of those that did not report significant result values analysed differences between groups and simply reported significance levels. Most of the studies analysed differences in attitudes between r/s groups (*n* = 22). Several studies reported only mean scores or percentages of various attitudes towards addiction and addiction treatment for differing r/s beliefs, rather than analysing differences between groups (Cuadrado, 2014, Cuadrado and Lieberman, 2011, Flórez et al., 2015, Hatchett et al., 2011). Five studies used correlational analyses between their measures of religion/spirituality and attitudes towards addiction. Generally, these correlational results suggest that higher religiosity is related to more negative attitudes towards addictions. Eleven studies utilized regression analyses to understand the relationship between religion/spirituality and attitudes towards addiction and addiction treatment.

### 3.3 Attitudes towards addiction by religion/spirituality

Five studies did not focus on a specific population, but rather used general nationwide or unspecified samples. In one such study, there were no significant differences between different forms of Christian religious affiliation, categorized as Catholic, “Ecclesia” (Episcopalian, Presbyterian, and Congregationalist), “Denominations” (Lutheran, Methodist, Baptist, and Disciples of Christ), and “Fundamentalists” (Holiness, Pentacostal, and Adventist), in terms of attitudes towards “alcoholics, alcoholism, and the treatment of alcoholism” (Linsky, 1965). However, the authors found that “Ecclesia” appeared to have significantly more positive attitudes than the other r/s affiliations in post-hoc tests. Conversely, another study found that those who rated their religiousness as very important or attended services more than once a week agreed that “most people who drink and drive are alcoholics or problem drinkers” (Lucchetti et al., 2014), yet, this study did not contrast different religious groups.

In terms of support for different models of addiction, higher religiosity and Christian affiliation appeared to be related to more support of the moral model of addiction. On a continuum between the moral and disease models, Christian individuals most supported the belief that alcohol use disorder is a moral issue and Jewish individuals most supported the belief that it is a disease problem, while Muslim individuals did not significantly differ from either of these (Weiss & Moore, 1992). Broadus and Evans (2015) found that, in an online sample, the importance of religion also predicted stronger belief in the moral model and weaker belief in the nature model, but did not predict belief in the psychology, sociology, or disease models. Further, religious service attendance did not predict belief in any of the models of addiction.

**Attitudes of Those Seeking Treatment.** In a sample of outpatient individuals completing treatment for alcohol or crack cocaine dependence in Brazil, religious practice did not predict internalised stigma for substance dependence (Santos da Silveira et al., 2018). In other words, religious practice did not predict negative attitudes towards the individual’s own substance use disorder.

**Provider Attitudes.** In studies looking at provider attitudes and religion/spirituality, three studies found that belief in the disease model of addiction, measured using the Addiction Belief Scale, was related to higher religion/spirituality scores on the Spiritual Belief Scale in clinicians from Australia, Canada, the UK, and the USA (Russell et al., 2011, Schaler, 1996, Schaler, 1997). Higher scores on the Spiritual Beliefs Scale also negatively predicted the belief that addiction is a choice but did not have a significant relationship to the belief that addiction is a way of coping (Russell et al., 2011). However, results presented in Schaler’s (1997) paper also showed that religious affiliation did not predict scores on the Addiction Belief Scale.

In contrast to the above findings, mean comparison analyses found that clinicians working in more secular organizations more strongly endorsed the disease, brain disease, and genetically predisposed causes of addiction compared to clinicians working in more faith-based organizations, who more strongly endorsed the religious model and the belief that a lack of parental bond causes addiction (Chu & Sung, 2014). Notably, Protestant affiliation predicted less belief in the disease model, but not a stronger belief in the religious model, whereas high self-perceived religiosity predicted belief in the religious model but not the disease model. Conversely, Hshieh and Srebalus (1997) found that those who identified as religious or spiritual had a stronger belief in the disease concept of addiction than those that identified as not having r/s beliefs. Additionally, in a sample of nursing faculty, Jewish participants, in comparison with participants of other religious affiliations, agreed more that nurses with chemical dependency problems are best understood as suffering from an illness (Bugle, Jackson, Kornegay, & Rives, 2003).

Other factors may interact with religion/spirituality’s relationship to attitudes towards addiction among treatment providers as well. In one of the few included studies attempting to manipulate a factor which may influence attitudes in some way, Hecker, et al. (1995) presented clinical members of the American Association of Marriage and Family Therapy one of four randomly selected vignettes about an individual (married monogamous female, married monogamous male, single female with varied partners, or single male with varied partners) engaging in a sexual interaction which met some, but not all, criteria for sex addiction according to Carnes (1983). The authors did not find that the vignette given interacted with religiosity to change attitudes. However, those with higher religiosity were more likely to perceive this client as a “sex addict” than those with low religiosity. Men with high religiosity perceived the vignette character as having more sexual pathology than men with low religiosity and women with high or low religiosity. Finally, one study noted that Catholic nurses who reported feeling uncomfortable providing care to those with alcohol use disorder had less favourable attitudes towards alcohol use disorder than non-sectarian individuals or Catholics who were comfortable providing such care (Gurel & Spain, 1977).

***Student Provider Attitudes.*** It appears that in student provider samples, the substance/behaviour in question may affect beliefs about addiction. In samples of medical, nursing, and social work students in Russia and Israel, there were no differences between religious and secular students in terms of beliefs about the addictive nature of cannabis, but there were differences in terms of how it should be used, with secular individuals being more supportive of legalization and medical use (Edelstein et al., 2020, Gritsenko et al., 2020). Crothers and Dorrian (2011) also found no significant differences between r/s affiliations or service attendance in terms of attitudes towards alcohol use disorder, using three different measures of attitudes (Marcus Alcoholism Questionnaire, Seaman-Mannello Nurses’ Attitudes toward Alcoholism Scale, Shortened version of the Alcohol and Alcohol Problems Perception Questionnaire) in nurses at a teaching hospital in Australia. However, among social work students, religiosity significantly predicted beliefs in the addictiveness of pornography and the view that pornography is a health issue, and belief in the addictiveness of pornography significantly predicted beliefs that pornography is a public health issue (Droubay & Butters, 2020).

***Clergy Attitudes.*** In many cultures and communities, clergy and religious leaders are seen as providers of treatment or first-line support for mental health problems, including addiction. Christian clergy on the Texas/Mexico border appeared to agree, more than disagree, that alcohol abuse was a reaction to stress in life (69%), a learned behaviour (85%), a moral problem (76%), and caused by peer pressure (65.5%; Hatchett et al., 2011). These results suggest that while the clergy generally do not see alcohol abuse as a problem of weak willpower and are split on their view of it being a genetic problem, they do see it as a moral failing influenced by the individual’s context.

**Adolescent Attitudes.** In the only included study focused on adolescents, Globetti, Alsikafi, & Christy (1977) reported that students at American military schools in Europe who frequented church less often had more permissive attitudes towards excessive drinking, which the authors equate to alcohol abuse.

**Undergraduate Student Attitudes.** Similar to other samples, there appears to be variability in whether there is a relationship between religion/spirituality and attitudes toward addiction in undergraduate samples. Acceptance of alcohol and alcoholism was associated with lower spirituality, religiosity, God consciousness, and less formal religious practices in India (Sukhwal & Suman, 2013). Jolly and Orford (1983) also found those who were student members of a British Christian Union had more negative attitudes towards alcohol use disorder than those who were not members of the Christian Union. However, there was no difference in attitudes towards alcohol use disorder or those with an alcohol use disorder between levels of religious observance, regardless of whether the questionnaire appeared to be focused on “normal” or problem alcohol behaviour. Moreover, Pedersen and Von Soest (2015) found that Christian affiliation predicted the belief that cannabis, but not alcohol, was harmful, including leading to dependence, compared to those with no religious affiliation in Norway. However, affiliation with Islam or another religion did not predict views that cannabis or alcohol was harmful.

**Religious/Spiritual Sample Attitudes.** Even in samples recruited by religion/spirituality there appeared to be variability in the relationship between religion/spirituality and attitudes. Loewenthal, et al. (2003) found that Protestants agreed more that alcoholics drink because they want to, compared to Jewish participants, but there was no difference in perceptions that “alcoholism is an illness” or that alcoholics need to quit to recover.^5^ Conversely, stronger belief that substance misuse would anger the creator and was religiously prohibited was related to the belief that misuse leads to addiction and disagreement with the belief that misuse is a normal stress reaction in Muslim students and clinicians in Kuwait (Bilal et al., 1990). Moreover, Flórez, et al. (2015) suggest that African American and Latino Christians appear to have a slight stigma against those with substance use disorder, based on their score being above the midpoint on a modified Alcoholism Stigma Scale, which focuses on moral problems with drug abuse.

### 3.4 Attitudes towards addiction treatment by religion/spirituality

Some studies looking at attitudes towards the treatment of addictions focused on attitudes towards certain treatment modalities. Extending their paper beyond attitudes towards addiction, Linsky (1965) found that those categorized as “Ecclesia” or “Denominations” preferred more scientific treatment for alcohol use disorder, but a significant minority of the “Denominations” group believed will-power and religious help was also effective. Catholics also typically preferred scientific treatment, particularly medical rather than psychological or psychiatric help, whereas the “Fundamentalists” group preferred religious help followed by the use of will-power.

Other research focused on attitudes towards public policy as a way of understanding attitudes towards those with an addiction, but these papers often conflated policy changes for use with those for addiction. In a nationwide sample from Brazil, agreement with increasing financial support for alcohol treatment programs did not differ by importance of religion and service attendance (Lucchetti et al., 2014). However, affiliation, importance of religion, and service attendance was related to belief in changing other public policies for alcohol use, such as an increase in the minimum legal age for alcohol sales or a ban on alcohol sales at food stores. Moreover, Protestants and Catholics reported lower tolerance of social policy change towards liberalisation of narcotics, such as the belief that prescribers who have a substance use disorder should be heavily punished and have their licenses removed, compared to other, mostly Jewish, religiously affiliated individuals (Rooney & Gibbons, 1966).

**Attitudes of Those in Treatment.** It appears that attitudes towards 12-step groups may be less related by religion/spirituality than inclusion of religion/spirituality in therapy in those seeking treatment, whose perceptions about important aspects of therapy appear to differ from providers. Two studies, focused on understanding feedback from patients in an inpatient treatment centre which was founded by an Episcopalian pastor (Daytop), found that preference for and acceptance of spiritually-based therapeutic community treatment was related to a higher frequency of prayer/meditation and stronger self-identification as spiritual (Dermatis et al., 2004, Dermatis et al., 2010). Higher spirituality, assessed by Adapted Intrinsic/Extrinsic Scale for Spiritual Orientation, also predicted more acceptance of, but not preference for, the therapeutic community principles (Dermatis et al., 2010). Results from the 2004 paper also showed that those who reported a higher belief in God more strongly preferred spiritually based interventions than those who did not, but no such difference was found in preference for the inclusion of 12-step groups. Religious affiliation predicted client’s willingness for a counsellor to include religion/spirituality in sessions, but not if the counsellor is knowledgeable about their religion/spirituality and has a different personal religion/spirituality or if the counsellor is not knowledgeable and has the same religion/spirituality (Diallo, 2013). Despite not explicitly testing the relationship between religion/spirituality and attitudes, Goldfarb, et al. (1996) found that students in an inpatient hospital unit reported being less spiritual or religious than dually-diagnosed patients on the unit and the authors discussed their results of attitudes towards treatment in terms of this difference in religion/spirituality. They found that patients more strongly endorsed the importance of inner peace, medical services, belief in God/Higher Power, outpatient treatment, AA meetings, a strong spiritual orientation, a supportive community or place of worship, and trusting people for treatment than the students did. Patients also ranked government benefits, inner peace, medical services, AA meetings, and a strong spiritual orientation higher than the students’ perceptions of the patients’ attitudes towards these same things. Conversely, the Religious Beliefs and Behaviours Scale scores of patients from two inpatient substance use units in a British hospital did not differ between those who rated their attitudes towards AA and NA as hostile, neutral, or positive (Best et al., 2001).

**Provider Attitudes.** Overall, religion/spirituality did appear to impact providers’ views of various treatments for addiction, with more religious individuals reporting more positive views towards spiritually based and 12-step programs (Chu and Sung, 2014, Galanter et al., 1991, Hshieh and Srebalus, 1997, Lawrence et al., 2012). Further, another study found that non-medical staff at a non-residential substance use disorder treatment centre who had a positive attitude towards AA and NA and reported being more likely to recommend patients to AA and NA scored significantly higher on the Spiritual Beliefs Questionnaire than those who did not (Day, Gaston, Furlong, Murali, & Copello, 2005). Nevertheless, there continued to be some variability in terms of which aspect of religion/spirituality is related to these attitudes. For example, members of a religiously fundamentalist dental and medical organisation believed that individuals from a non-believing Christian background would benefit more from AA than committed Christians, who they believed would benefit more from prayer (Galanter, Larson, & Rubenstone, 1991). In contrast, another study found no differences in attitudes towards various treatments for alcohol use disorder between physicians and psychiatrists that were given a vignette about a Christian client who attended church and those given a vignette of a client who did not (Lawrence et al., 2012). In a sample of nursing faculty, Bugle, et al. (2003) found some differences in beliefs about how nurses with chemical dependency should be treated between religious affiliations.^6^ Finally, male members of the American Association of Marriage and Family Therapy with low religiosity perceived that a vignette character with potential “sex addiction” needed fewer sessions of therapy than female members with low religiosity, and males with high religiosity perceived that therapy would require more sessions than females with high religiosity (Hecker et al., 1995).

***Clergy Attitudes.*** Two studies found that most Catholic priests from Florida and the USA-Mexico border were willing to witness a *juramento* (Cuadrado, 2014, Cuadrado and Lieberman, 2011), even if they did not believe it was effective as a form of treatment for alcohol use disorder (80%: Cuadrado, 2014).^7^ Most priests believed the ritual to be somewhat effective (67%: Cuadrado, 2014), while 25% reported being unsure and 8% reported believing it was not effective. In another study using a sample of clergy, most believed that individuals should not be punished and reported that they knew places to refer others for treatment (Hatchett et al., 2011).

**Religious/Spiritual Sample Attitudes.** Stronger belief that substance misuse would anger the creator and is religiously prohibited was related to a belief in the importance of offering help to the user in Muslim students and clinicians in Kuwait (Bilal et al., 1990).

### 3.5 Differences by religious affiliation

Of the 26 studies which reported religious affiliation, fourteen studies did not report any analyses of differences between religious affiliations and one study only reported that there was a difference (Diallo, 2013). While most of the studies utilized Christian samples, seven studies used entirely Christian samples and three of these discussed their findings regarding attitudes with the assumption that these were specifically views of Christian individuals (Cuadrado, 2014, Hatchett et al., 2011). Protestant individuals were found to be less supportive of the disease model and more supportive of increasing taxes for alcohol compared to non-Protestant individuals (Chu and Sung, 2014, Lucchetti et al., 2014), but not significantly different to other Christians in terms of attitudes towards addiction treatment or those with an addiction (Linsky, 1965). Non-evangelical protestants were seen to support participation in AA, view spirituality as critical for treatment, more likely to refer to faith based treatments, and be less supportive of pharmacotherapy than some other affiliations, such as Jewish or unaffiliated individuals (Lawrence et al., 2012). One study found that Catholic individuals had generally more negative attitudes towards those with alcohol use disorder (Gurel & Spain, 1977). Jewish individuals appeared to most strongly agree with the disease/illness model of addiction or more strongly disagree that it is a moral weakness compared to Christian groups (Bugle et al., 2003, Loewenthal et al., 2003, Weiss and Moore, 1992). Finally, three studies found relatively similar attitudes between r/s affiliations studied (Pedersen and Von Soest, 2015, Rooney and Gibbons, 1966, Schaler, 1997).

### 3.6 Differences by substance or behaviour

**Substances.** Most studies found similar relationships between religion/spirituality and attitudes to various substances or measured attitudes towards addiction to several substances together. However, Christians appeared to view cannabis as more harmful and secular individuals appeared to more strongly support legalization and medical use of cannabis (Edelstein et al., 2020, Gritsenko et al., 2020, Pedersen and Von Soest, 2015).

**Behaviours.** Of the four studies which looked at attitudes towards behavioural addictions, two included various behaviours as potentially addictive when assessing general attitudes towards addiction (Broadus and Evans, 2015, Russell et al., 2011). The results of the other two studies suggested that higher religiosity was related to an increased likelihood of perceiving an individual who has some symptoms of “sex addiction” as a “sex addict”, perceiving that the individual requires more treatment, and the belief that pornography is addictive (Droubay and Butters, 2020, Hecker et al., 1995).

## 4 Discussion

Addiction is an enduring part of the human condition that, like most aspects of the human experience, has been discussed by religious leaders and institutions for millennia. Religious beliefs, institutions, and persons remain important influencers of the addiction treatment process, and, in many cases, the policy-making processes that dictate addiction treatment. At the outset of the present work, we proposed to conduct a scoping review of empirical literature examining the relationship between r/s beliefs and attitudes towards addiction and addiction treatment. Below, we briefly summarize the above findings and discuss the implications of these results, including the difficulty in comparing studies to find clear conclusions.

Overall, reviewed studies reported mixed and, at times, contradictory findings. Most studies reported a non-significant relationship between one of their religion/spirituality measures and an attitude measure (*n* = 23). Although the absence of such links is not necessarily evidence that no such links exist, it does suggest that current literature may not adequately assess the variety of ways that religion might influence attitudes toward addiction and treatment. Among the significant results reported, there is some evidence that higher religiosity and adherence to r/s practices is related to more belief in the disease model of addiction (*n* = 6), negative attitudes towards a substance use disorder or behavioural addiction (*n* = 7), and a stronger belief that treatments, particularly spiritually based treatments (*n* = 6), are needed and will help an individual with these diagnoses (*n* = 10). Moreover, religion/spirituality appeared to be more related, in terms of more significant results, to attitudes about addiction treatment than attitudes about addiction itself. That is, there was more evidence to support the conclusion that religion/spirituality are associated with attitudes about treatment than there was to support the conclusion that religion/spirituality is linked to attitudes toward addiction itself.

Across included papers, there were a high variety of measures used for both attitudes and religion/spirituality. In the studies which used multiple religion/spirituality measures, results varied within studies, suggesting that variability in results may be due to measurement problems. Further, results were very similar in three studies which used the same measures (Russell et al., 2011, Schaler, 1996, Schaler, 1997), suggesting again that the measures used may have impacted results. These results are consistent with those of previous reviews noted above (e.g., El Khoury et al., 2019, Geppert et al., 2007, Kinder, 1975). In short, poor-quality measurement has likely impaired research in this domain.

Most of the studies focusing on specific populations, such as undergraduate students, appeared to suggest uniform trends. However, in provider and student provider samples it appeared that other factors, such as the sex of the respondent or the substance/behaviour in question, may interact with religion/spirituality in relation to attitudes towards addiction. Those seeking treatment appeared to differ in their perceptions about which aspects of therapy are important and religion/spirituality appeared more related to attitudes towards the inclusion of religion/spirituality in therapy than towards 12-step groups. However, in the one study on attitudes towards addiction in adolescents, no relationship was found between religion/spirituality and attitudes. Results from clergy samples appeared to suggest that they view addiction as a moral failing influenced by the individual’s context and studies focused on their willingness to use a religious ritual as a method of treating addiction.

A minority of studies (*n* = 4) focused on attitudes toward behavioural addictions, and these results appear to be relatively similar to those of substances, which primarily focused on attitudes towards alcohol. However, only two of the behavioural studies looked at the behaviour specifically rather than attitudes towards addiction generally. More research in this area is particularly needed given evidence from prior works that r/s beliefs may impact value and moral judgements regarding certain behaviours, such as pornography, which may in turn impact attitudes towards the addiction itself or its treatment (e.g., Grubbs et al., 2019, Grubbs et al., 2020, Grubbs et al., 2020, Grubbs et al., 2020, Grubbs and Perry, 2019). As suggested in previous works (Royce, 1985), specific religious affiliation (in contrast with general religiousness) appeared to have some impact on attitudes towards addiction, particularly treatment. When comparing r/s affiliations, Jewish individuals appeared to support the disease model more strongly, and Christian groups, particularly Protestant and evangelical Protestant individuals, more strongly supported spiritually based treatments. There did not appear to be many clear differences in the relationship between religion/spirituality and attitudes between countries, with the majority of studies focused on the USA; however, in the one study which statistically compared countries, higher religion/spirituality predicted stronger belief in addiction as a choice in the UK but not the USA (Russell et al., 2011).

### 4.1 Implications and further directions

Many of the studies screened out for this review (*n* = 652) and several of the reviews previously conducted (e.g., Karaga et al., 2016, Seva Diaz and Vazquez Casabona, 1975) focused on attitudes towards substance use and behaviours, but not attitudes towards addiction to those substances or behaviours. That is, most prior work has only examined how religious individuals view using substances or engaging in potentially addictive behaviours, rather than attitudes toward addiction to those things. As such, we have relatively little knowledge about how religiousness may influence whether people think something is addictive, how they view that addiction, and, perhaps most importantly, how they view people who are dealing with such addictions.

The results from this review suggest that the relationship between religion/spirituality and attitudes towards addictions vary considerably and overall conclusions are difficult to draw due to the varying methodology used. There is much still unknown about this relationship, given that many of the results in this review were non-significant or varied in terms of other factors, such as sex of the respondent. These potential moderating factors, along with the specific aspects of religion/spirituality, must be systematically evaluated to understand this relationship more fully. More importantly, comparing these studies proved difficult given the large variety of measures and methods used; thus, all conclusions are general and point to an area in need of clearer methodological research.

Only eight of the studies looked at a specific model of addiction and attempted to understand whether views about addiction conform to known theories regarding addiction. In most cases, the analysis or data between religion/spirituality and attitudes was a secondary focus in the analyses and simply an attempt to understand potential covariates in the article. As such, in many cases this relationship was not the main focus of the articles and so was not fully developed. A better theoretical underpinning for such relationships should be created and expanded upon, so that research may be better guided in terms of what specific aspects of these variables should be analysed. Further, researchers and publications should allow full reporting of results, including covariates measured, to aid reviews and readers in synthesizing the data, perhaps in supplementary materials.

Similarly, in many cases articles relied on unvalidated or single item measures of both religion/spirituality and attitudes towards addiction and addiction treatment, such as religious affiliation, service attendance, or agreement with a statement about the cause of addiction. Such reliance likely factored into the high variability in results because each measure appears to assess slightly different aspects of each of these variables. In this way, it is difficult to gain a clear view of the potential relationship between religion/spirituality and attitudes. Prior research suggests that each of these measures assesses different aspects of religion/spirituality and notes the importance of differentiating attitudes between those who identify as religious or spiritual (Zwingmann, Klein, & Büssing, 2011). Nevertheless, as previously noted, some studies which used the same measures did appear to have similar significant results (Russell et al., 2011, Schaler, 1996, Schaler, 1997); although two of these articles seemed to use the same sample. As such, it is imperative that future researchers fully operationalize these variables, ensure that their measures are valid and reliable, and fully explore this relationship so that clearer conclusions and comparisons can be made in the future.

Moreover, many of the included studies neglected to report religious affiliation when discussing the relationship between religion/spirituality and attitudes. As previously noted, there are significant differences between religions and denominations in terms of their views of substances and how those with addictions should be treated (e.g., Royce, 1985). Therefore, such an omission makes these studies difficult to interpret fully. Future researchers should ensure that this demographic variable is measured and reported, along with fully reporting other variables and result statistics.

All the studies included in this review utilised cross-sectional research designs. The lack of longitudinal designs means that no conclusions about causal relationships can be made. As previously noted, attitudes and religiosity may change across the lifespan (e.g., Bengtson et al., 2015, Chan et al., 2015) and there might be several factors which may influence an individuals’ attitudes (e.g., Hecker et al., 1995). Therefore, future researchers should attempt to employ longitudinal research designs, potentially across the lifespan, to fully understand this relationship. Moreover, much of the research focused on the self-report of participants using quantitative measures. Future research may benefit from examining individual’s responses to those with an addiction, such as looking at clinician’s referral rates. Further, while this review focused on quantitative research, future studies should utilize qualitative analysis to better understand how attitudes and religion/spirituality may be related. Similarly, there appears to be a lack of research in non-Western countries, and particularly in individuals who are not Christian or from an Abrahamic faith. Prior works have explored how religion/spirituality may be important factors when looking at differences in views of substances/behaviours between countries, such as China and the USA (Pontell & Geis, 2008), and how factors such as race may interact with attitudes (Oler, 1996, Perryman, 2020). Future research would benefit from expanding this research to other demographics and geographical areas.

In a similar way, it is possible that public policies are swayed by the country or policymaker’s religion/spirituality. Even though no included studies measured this explicitly, one example of this may be the prohibition movement in the USA (Schmidt, 1995). Other qualitative research has also suggested this relationship exists by studying the views of harm reduction interventions by stakeholders in substance use treatments (Philbin et al., 2008, Philbin et al., 2009, Szott, 2020). However, the present review did not find many clear differences in the relationship between religion/spirituality and attitudes toward addiction between countries, perhaps due to a lack of research in non-US countries and between country comparisons.

Prior research is split on the impact of religiosity as a protective factor for addiction, with some studies suggesting that it does not impact things such as delay discounting (Thornton, McCarty, & Stokes, 2017) while others suggest it may be an important factor which could be utilized for treatment (Seghatoleslam et al., 2015). The results of the present review are also conflicted and nuanced making it difficult to draw specific clinical recommendations at this time. As such, a primary recommendation until further research is completed must be to take an open and exploratory stance, personally and interpersonally, towards the influence of different aspects of religion/spirituality on beliefs about addiction and addiction treatment in clinicians, policy makers, and those with an addictive disorder. Such a stance would aid in reducing any potential biases which may be present and support the appropriate use of religion/spirituality in treatment, based on a client’s needs and own beliefs. Meanwhile, it is imperative that more research is done to fully understand the impact of religion/spirituality on providers’ attitudes toward addiction and how religion/spirituality may impact policy decisions surrounding the treatment of those with addictions.

Clearly, the state of research in this area in terms of methodology has several major problems, many of which have been detailed about research in psychology in general as well, with particular concerns surrounding applied psychology (e.g., Flake and Fried, 2020, Grubbs, 2021, Shrout and Rodgers, 2018). Such methodological concerns are particularly problematic in this area given the large number of addiction treatment programs run by or based on a r/s tradition (e.g., AA or Celebrate Recovery). Our lack of clear understanding about how religion/spirituality may be related to views about addiction leaves us unable to recognize how religion/spirituality may be impacting the treatment of those with an addiction, personally, clinically, or societally. Without this knowledge we can neither face and combat potential biases about addiction from this domain nor can we utilize this domain in supporting the lives of those with an addiction or work with r/s communities effectively to care for such groups. Therefore, it is imperative that future research in this area, even when this relationship is not a main focus, pay close attention to ensuring rigorous, carefully operationalized, and theory driven methodology is used.

## 5 Conclusion

Addiction is a common phenomenon that likely intersects with religious belief and practice in numerous ways. Not surprisingly then, religious beliefs likely influence how people think about addiction. Expanding on previous reviews, the present work notes that there is wide variability in the relationship between individual religion/spirituality and attitudes toward addiction and addiction treatment, for a variety of reasons. Nevertheless, in general, higher religiosity appeared to be related to more negative views toward addictions and the belief that such addictions should be treated by more spiritually based treatments. Despite this, current research remains inconsistent and unclear to such an extent that a full understanding of the influences of religion/spirituality on attitudes is allusive, despite clear evidence that religion and spirituality are likely consequential in influencing such attitudes. Future research is necessary to fully explain the nuances between various aspects of religion/spirituality and attitudes towards addiction, any potential causal links between these variables, how this may affect the therapeutic relationship or policy makers’ decisions, and to understand the potential benefits or drawbacks from such a relationship.

**Role of funding sources**

This research did not receive any specific grant from funding agencies in the public, commercial, or not-for-profit sectors.

**Funding**

This research did not receive any specific grant from funding agencies in the public, commercial, or not-for-profit sectors.

### CRediT authorship contribution statement

**Jennifer T. Grant Weinandy:** Conceptualization, Methodology, Investigation, Formal analysis, Data curation, Visualization, Project administration. **Joshua B. Grubbs:** Conceptualization, Supervision.

## Declaration of Competing Interest

The authors declare that they have no known competing financial interests or personal relationships that could have appeared to influence the work reported in this paper.

## Footnotes

1 Behavioural addictions remain highly controversial within psychological and psychiatric research, with mental health research communities generally acknowledging that some behaviours can become dysregulated or compulsive but disagreeing whether or not such dysregulation represents true addiction or some other form of psychopathology (e.g., Griffiths, 2017; Kuss & Griffiths, 2015). Although such debates bear very strong implications for the conceptualization and treatment of out-of-control behaviour patterns, the present work does not seek to debate this particular issue. More specifically, for the present work we refer to behavioural addictions for the sake of parsimony, though we acknowledge that the status of such addictions is still subject to ongoing debate.

2 Religion or religiosity refers to a person following a set of beliefs and practices about their spiritual life prescribed by an institution (e.g., Christianity). Whereas spirituality refers to an internal belief or practice of an individual regarding the spiritual domain. In this way, an individual may be religious, spiritual, both or neither, and both terms refer to an individual’s thoughts and practices within the spiritual domain (Hill & Pargament, 2003).

3 British and American spellings of the word “behaviour” were searched in all cases where they are used.

4 While nicotine addiction remains a significant public health concern (e.g., Benowitz, 2010), it is often primarily treated in outpatient and primary care settings and has a wider range of treatment options available than other addictions.

5 It should be noted that there was some discrepancy between the tables and text in this paper: the text stated that Protestants agreed more that alcoholics drink because they want to rather than Jewish individuals.

6 The text and table in this article conflict somewhat, whereby the table states that Catholic individuals believe that a student should not be expelled and reported to the licensure board when there is evidence of impaired use, but the text states that Catholics do not significantly differ from the other groups and the other affiliated individuals believe this instead.

7 A *juramento*, according to the authors, is a religious ritual developed in Mexico in which the user commits to abstinence for a specified time by promising to remain abstinent to a saint, usually The Virgin of Guadalupe.
