# Supplementary material for: Religious and spiritual beliefs and attitudes towards addiction and addiction treatment: A scoping review
Source: Addict Behav Rep. 2021 Nov 14;14:100393. doi: 10.1016/j.abrep.2021.100393 (PMC8664870; doi:10.1016/j.abrep.2021.100393)
Supplement: Supplementary Data 1 [file mmc1.docx]

**Supplementary Table for *Religious and Spiritual Beliefs and Attitudes Towards Addiction and Addiction Treatment: A Scoping Review***

| Supplementary Table 1.  *Authors, assessment methods, country, sample, study design, and relevant findings of the relationship between religion/spirituality and attitudes towards addiction.* | | | |
| --- | --- | --- | --- |
| Author (Year); Country; Study Design; Sample Size; Sample Characteristics; Religious Affiliation; Addiction Studied | Attitude Measure and Religious/Spiritual Measure (Mean (SD) or Percentage of participants) | | Statistical tests; Relevant Findings |
| **Best, et al. (2001);** United Kingdom; Interview;  *N* = 200: Patients recruited from an inpatient alcohol unit and an inpatient drug use unit in one hospital;  Alcohol unit (*n* = 100); men (82%), women (18%); *M*age (SD) = 41.8 (9.9); Caucasian (89%), Black (4%), Other (7%);  Drug unit (*n* = 100); men (88%), women (12%); *M*age (SD) = 33.2 (7.6); Caucasian (85%), Black (14%), Other (1%);  Affiliation (NR);  Alcohol, heroin, & cocaine | Attitude: **Rating of attitude towards NA or AA** (1 = *Very negative*: 5 = *Very positive*):  Hostile (*Very or slightly negative*; 32.3%):  Alcohol unit (42%),  Drug unit (21.7%);  Neutral (*Neutral*; 31.2%):  Alcohol unit (31%),  Drug unit (29.4%);  Positive (*Very or slightly positive*; 37.5%):  Alcohol unit (27%),  Drug unit (48.9%)  R/S: **Religious Beliefs and Behaviour Scale** (Connors, Tonigan, & Miller, 1996; range = 0-30):  Alcohol unit = 5.1 (5.7);  Drug unit = 5.4 (5.7) | | ***F* test** between attitude groups in terms of r/s scores (*df* = 190) = 1.32 (ns; *p* = .27):  *Mean* (SD) for R/S by attitude group:  Hostile = 4.4 (5.2),  Neutral = 5.2 (5.7),  Positive = 6.0 (5.9) |
| **Bilal, et al. (1990)**; Kuwait; Paper questionnaire;  *N* = 1001: Muslim participants recruited from the only University in the country, two randomly selected schools, a general hospital, and five primary care clinics;  men (53.1%), women (46.9%); *M*age (SD) = 30.3 (13.8); Kuwaiti (47.4%), Other Arab (52.7%);  Muslim (100%);  Alcohol, drugs, & smoking | Attitude: **Arab-Muslim Attitude Questionnaire** (0 = *Disapprove*: 2 = *Approve*; range = 0-60):  Enlighted Attitude (importance of offering help to the deviant user):  Disapprove (3.8%), Unsure (5.3%), Approve (90.9%);  Punishing Attitude (wicked disbeliever should be punished by Islamic law):  Disapprove (7.4%), Unsure (66.6%), Approve (26%);  Drug misuse (Undesirable and may lead to addiction):  Disapprove (80.9%), Unsure (15.5%), Approve (3.6%);  Permissive Attitude (drug misuse is normal reaction to stress and smoking is civilized):  Disapprove (50.2%), Unsure (44.2%), Approve (5.6%)  R/S: **Religious Subscale of Arab-Muslim Attitude Questionnaire** (misuse is prohibited by religion and angers the creator; 0 = *Disapprove*: 2 = *Approve*; range = 0-60):  Disapprove (1%), Unsure (5.4%), Approve (93.6%) | | **Correlation** between Religious Attitude and:  Enlighted Attitude (*r* = .27***),  Punishing Attitude (*r* = .06, ns),  Drug misuse (*r* = .13***),  Permissive Attitude (*r =* -.09**) |
| **Broadus & Evans (2015): Study 3**; USA; online questionnaire;  *N* = 448: Participants recruited via InfoUSA and Survey International online platforms;  men (39.8%), women (60.2%); *M*age (SD) = 47.1 (15); Caucasian (87.5%), Hispanic (8.5%), Asian (2.5%), Black (6.6%), Indian/Alaskan Native (0.6%), Pacific Islander (0.4%), Native Hawaiian (0.4%);  Affiliation (NR);  Cocaine, alcohol, cannabis, & gambling | Attitude: **Public Attitudes about Addiction Scale** (1 = *Strongly disagree*: 5 = *Strongly agree*; range = NR): NR  R/S: **Importance of Religion** (range = 1-7) = 4.3 (2.2);  **Religious Service Attendance**: Never attended (46%), One or more times per week (19%) | | **Regression** predicting each of the Addiction Attitude subscales, alongside age, gender, income, race, marital status, education, employment, treatment history, whether they identified as an addict, and the area in which they lived:  Importance of Religion: Psychology (𝛽 = .11, ns), Moral (𝛽 = .17***), Nature (𝛽 = -.12*), Sociology (𝛽 = .10, ns), Disease (𝛽 = .10, ns);  Religious Service Attendance: Psychology (𝛽 = -.02, ns), Moral (𝛽 = -.05, ns), Nature (𝛽 = -.09, ns), Sociology (𝛽 = .08, ns), Disease (𝛽 = -.07, ns) |
| **Bugle, et al. (2003)**; USA; paper survey;  *N* = 324: Nursing faculty recruited from 5 randomly selected schools;  *M*age (SD) = 50 (NR);  Protestant (51.5%), Catholic (29.3%), Jewish (2.2%), Other (7.7%);  Chemical dependency | Attitude: **Adapted Perceptions of Student Nurse Impairment Inventory** (Hendrix, Sabritt, McDaniel, & Field, 1987; 1 = *Strongly disagree*: 5 = *Strongly agree*):  Top 5 disagreement:  Little can be done to help impaired student nurses (97.5%);  Help needed by impaired student nurses requires insight from other student nurses (95.7%);  Even after treatment, it is unusual for impaired students to be productive and trustworthy (94.1%);  The Board should publish name of license applicants who disclose a chemical dependency history (92.9%);  Impairment is usually caused by a weakness in the student nurse’s personality (92.3%);  Top 4 ambivalent (agreement):  Student nurse license applicants disclosing chemical dependency history should be issued a probationary license to assure public safety (51.8%);  A support group of other student nurses would help most impaired student nurses (45.1%);  I could, based on the student’s appearance or behavior, probably recognize an impaired student nurse (46.6%);  Student nurses suspecting impairment in a fellow student are responsible to help the student receive assistance (55.5%);  Top 5 agreement:  Student nurses should notify instructor if they suspect a student is impaired (93.8%);  Board should notify impaired applicants of their legal and due process rights (92.6%);  Instructors are responsible to help impaired students receive assistance (91.4%);  Difficulties encountered prior to nursing school most often cause student impairment (89.2%);  Board should provide assistance referral when license applicants disclose chemical dependency history (88.9%);  R/S: **Religious affiliation** | | **Chi-squared** responses by religious affiliation for each statement:  Catholics agreed most:  When a dean has concrete evidence that a student nurse is impaired, the dean has a responsibility to suspend the student, pending investigation of the charges (𝜒^2^ = 24.17, *p* = .004);  The State Board of Nursing should provide resources to support research on the prevention and treatment of impaired student nurses (𝜒^2^ = 24.05, *p* = .020);  Catholics disagreed most:  Impairment is a widespread problem among student nurses (𝜒^2^ = 55.87, *p* = .000);  “Other” (table states Catholic) disagreed most:  When a dean has concrete evidence that a student nurse is impaired, the dean has a responsibility to expel that student immediately and to inform the State Board of Nursing in case the student should ever apply for licensure (𝜒^2^ = 23.12, *p* = .006);  Jewish agreed most:  Impaired nurses can best be understood as people who suffer from an illness (𝜒^2^ = 17.31, *p* = .044);  The State Board of Nursing’s responsibility should include offering graduate nurses who disclose a history of chemical dependency referral to sources of assistance (𝜒^2^ = 18.21, *p* = .033) |
| **Chu & Sung (2014)**; USA; mailed survey;  *N* = 110: Randomly selected counsellors from the faith-based 2006 Teen Challenge Directory (*n* = 112) and the secular National Directory of Drug and Alcohol Abuse Treatment Programs 2005 (*n* = 105);  Faith-based counsellors (*n* = 59): men (61%), women (39%); *M*age (SD) = 40.73 (9.56); Caucasian (66%), Hispanic (9%), Black (20%), Native American (3%), Other (2%);  Protestant (56.9%), Other (43.1%);  Secular counsellors (*n* = 51): men (49%), women (51%); *M*age (SD) = 48.35 (10.76); Caucasian (75%), Asian (2%), Black (22%), Others (2%);  Protestant (28.6%), Judaism (4.1%), Hinduism (2%), Catholic (14.3%), Muslim (6.1%), Buddhism (2%), Agnostic (4.1%), Other (38.7%);  Drug abuse | Attitude: **Drug Abuse Counsellor Survey: Drug Abuse** (1 = *Best explanation*: 12 = *Worst explanation*):  Religious model: Faith-based = 9.27 (4.23), Secular (*n* = 49) = 5.10 (4.06);  Disease model: Faith-based (*n* = 58) = 7.41 (6.66), Secular (*n* = 49) = 16.61 (6.94);  Brain disease: Faith-based = 3.07 (3.50), Secular = 8.14 (4.45);  Lack of parenting/bond: Faith/based = 7.31 (3.67), Secular = 5.16 (3.47);  Genetically predisposed: Faith-based = 4.38 (3.88), Secular = 8.58 (3.42);  **Drug Abuse Counsellor Survey: Drug Abuse Treatment** (1 = *Least important*: 10 = *Most important*):  Addressing religious need: Faith-based = 10.32 (3.45), Secular = 8.59 (3.14);  Medication: Faith-based = 4.41 (3.01), Secular = 6.73 (3.29);  Positive self-concept: Faith-based = 8.71 (3.45), Secular = 9.98 (2.46);  Reduce stress: Faith-based = 7.41 (3.18), Secular = 9.08 (2.61);  R/S: **Protestant affiliation**;  **Faith-based vs Secular Employer**;  **Extent of Religiosity** (1 = *Not religious or spiritual at all*: 4 = *Very religious or spiritual*; range = 1-4):  Faith-based (*n* = 58) = 3.83 (.38): Moderately religious (17.2%), Very religious (82.8%);  Secular = 3.39 (.72): Not religious at all (2%), Slightly religious (7.8%), Moderately religious (39.2%), Very religious (51%) | | ***t* test** for mean comparison between Faith-based and Secular counsellors:  Religious model = 5.20***,  Disease model = -6.98***,  Brain disease = -6.65***,  Lack of parenting/bond = 3.10**,  Genetically predisposed = -5.92***,  Addressing religious need = 2.74*,  Medication = -3.86***,  Positive self-concept = -2.19*,  Reduce stress = -2.98**;  **Multiple regression** predicting belief in each cause of addiction, alongside gender, age, race, education, certification, years of experience, caseload, and whether the individual was recovering (𝛽):  Religious Model:  Faith-based vs Secular = 2.867*,  Protestant vs Non-Protestant = -.038 (ns),  Extent of Religiosity = 1.545*  Disease Model:  Faith-based vs Secular = -5.684**,  Protestant vs Non-Protestant = -4.076**,  Extent of Religiosity = -.727 (ns)  Addressing Religious Needs:  Faith-based vs Secular = .911 (ns),  Protestant vs Non-Protestant = .804 (ns),  Extent of Religiosity = 1.590*  Medication:  Faith-based vs Secular = -1.081 (ns),  Protestant vs Non-Protestant = -.306 (ns),  Extent of Religiosity = .085 (ns) |
| **Crothers & Dorrian (2011)**; Australia; questionnaire;  *N* = 49: Nurses at a teaching hospital were recruited from four wards and an educational seminar;  women (92%), men (8%); *M*age = 39 (11);  Identify with a religious denomination (65%);  Alcohol | Attitudes: **Marcus Alcoholism Questionnaire** (Marcus, 1980; 1: *completely disagree*; 7: *completely agree*; higher score related to more negative attitude):  Prognosis for recovery = 3.01 (1.37),  Alcoholism and character defect = 2.59 (1.22),  Social status of the alcoholic = 2.91 (1.02),  Alcoholism as an illness = 3.13 (1.67; scoring most negative attitude score = 4.2%);  **Seaman-Mannello Nurses’ Attitudes toward Alcoholism Scale** (Seaman & Mannello, 1978; 1: *completely disagree*; 5: *completely agree*; higher score related to more positive attitude):  Personal/Professional satisfaction = 3.17 (1.27; scoring most negative attitude score = 12.5%),  Inclination to identify (helpable) = 4.17 (.982),  Personal attitudes toward drinking = 4.56 (1.15);  **Shortened version of the Alcohol and Alcohol Problems Perception Questionnaire** (Anderson & Clement, 1987; 1: *completely disagree*; 7: *completely agree*; higher score related to more positive attitude):  Work satisfaction = 3.66 (1.11; scoring most negative attitude score = 8.2%),  Role adequacy = 4.04 (1.33; scoring most negative attitude score = 4.1%),  Role legitimacy = 4.87 (1.14; scoring most negative attitude score = 2%),  Task specific self-esteem = 4.96 (1.22; scoring most negative attitude score = 16.7%),  I want to work with drinkers = 3.4 (1.23; scoring most negative attitude score = 14.3%),  Pessimism is the best attitude to take toward drinkers = 2.85 (1.4; scoring most negative attitude score = 2%);  R/S: **Religious affiliation**;  Of those who identify with a denomination: **Service attendance**:  Never (26%), Once or twice a year (32%), More than twice a year (40%) | | **ANOVA and group *t*-tests** between r/s variables in terms of attitude:  Marcus Alcoholism Questionnaire = NR (ns),  Seaman-Mannello Nurses’ Attitudes toward Alcoholism Scale = NR (NR),  Shortened version of the Alcohol and Alcohol Problems Perception Questionnaire = NR (ns) |
| **Cuadrado & Lieberman (2011)**; USA; mailed survey;  *N* = 162: Priests in parishes located in counties where at least 10% of the population was Hispanic according to the US Census 2004 in Florida volunteered;  men (100%); Hispanic (40.3%);  Roman Catholic (100%);  Alcohol | Attitude: **Willingness to witness a *juramento***^1^: Yes (75.2%), No (24.8%)  R/S: **Religious Affiliation** | | **Percentage**: Catholic priest’s willingness to witness a *juramento*:  Yes (75.2%), No (24.8%) |
| **Cuadrado (2014)**; USA; mailed survey;  *N* = 196: Priests in parishes located in counties where at least 10% of the population was Hispanic according to the US Census 2004 along the US-Mexico border volunteered;  men (100%); Hispanic (39%);  Roman Catholic (100%);  Alcohol | Attitude: **Perception of the effectiveness of *juramentos*** (1 = *Not, Somewhat, or Less than half the time;* 2 = *If with self- help programme, followed by treatment, family is committed to helping, or the person’s faith is deep*; 3 = *Yes in majority of cases, for time pledged, or for long periods of time if pledge is renewed*; 4 = *do not know or did not follow-up on person’s situation*; *n* = 72):  Not very or not at all (1) = 8.3%,  Yes, with conditions (2) = 38.9%,  Yes, without conditions (3) = 27.8%,  Do not know (4) = 25%;  **Willingness to witness a *juramento***:  Yes (79%), No (21%)  R/S: **Religious Affiliation** | | **Percentage**: Perception of the effectiveness of *juramentos* in Catholic priests (*n* = 72):  Not very or not at all = 8.3%,  Yes, with conditions = 38.9%,  Yes, without conditions = 27.8%,  Do not know = 25%  Catholic Priest’s willingness to witness a *juramento*: Yes (79%), No (21%);  Of those who have previously witnessed one (*n* = 72):  Yes (97.1%), No (2.9%);  Of those who did not believe it was effective (*n* = 6):  Yes (80%), No (20%) |
| **Santos da Silveira, et al. (2018)**; Brazil; Interview;  *N* = 461: Volunteers beginning outpatient treatment for crack cocaine and/or alcohol dependence at the Psychosocial Care Center for Alcohol and Drugs at Juiz de Fora;  Age = 18 years old or more; men (97.1%), women (8.3%);  Affiliation (NR);  Substance dependence | Attitude: **Brazilian version of the Internalized Stigma of Mental Illness Scale adapted for Substance Dependence** (Ritsher, Otilingam, & Grajales, 2003; range = 34-93): 67.6 (8.82);  R/S: **Religious Practice**: Yes (55.4%), No (44.6%) | | **Multiple regression** predicting Internalized Stigma:  With gender, unemployment, education, substance used, involvement in illicit activities, and whether they live with someone:  Religious Practice (𝛽 = .043, ns)  With self-esteem, hope, depression, gender, unemployment, education, substance used, involvement in illicit activities, and whether they live with someone:  Religious Practice (𝛽 = .078, ns) |
| **Day, et al. (2005)**; UK; mailed questionnaire;  *N* = 347: Non-medical staff at non-residential SUD treatment centres sent the questionnaire after their name was given by the centres;  Women (63%);  Affiliation (NR);  Alcohol & drugs | Attitude: **Rating of attitude towards NA or AA** (Best et al., 2001): Positive or Very Positive (39%), Neutral (54.8%);  **Recommend attendance at AA or NA to clients**: Likely or Very likely (45.5%):  Nursing staff (n = 60, 69.8%),  Drug workers/counsellors (n = 75, 35%)  **Adapted Comprehensive Drinker Profile** (Miller & Marlatt, 1984; *n* = 225): Bad habit (78%), Disease (22%)  **Number of steps agreed, or partially agreed, with** (Strongly Agree/Agree vs Strongly Disagree/Disagree per step):  Personal Responsibility = 3.16 (2.07):  Step 1 (46.6 vs 44.3%), Step 4 (56 vs 20.7%), Step 8 (54.6 vs 25%), Step 9 (58.8 vs 16.7%), Step 10 (70 vs 13.1%), Step 12 (29.5 vs 41.7%);  Higher Power-mediated = 1.73 (2.12):  Step 2 (27.1 vs 55.7%), Step 3 (25.6 vs 58%), Step 5 (45.2 vs 35.2%), Step 6 (29 vs 61.8%), Step 7 (22.3 vs 60.8%), Step 11 (34.2 vs 44.5%)  **Belief that** (Strongly Agree/Agree vs Strongly Disagree/Disagree):  AA/NA encourages honesty and self-awareness (53.2 vs 17.9%),  AA/NA encourages taking responsibility (38.6 vs 38.6%),  the 12 steps put clients off meetings: (52.4 vs 11.9%),  having a sponsor through AA/NA would be helpful (37.7 vs 11.4%);  R/S: **Spiritual Beliefs Questionnaire** (Christo & Franey, 1995; range = 7-35) = 22.73 (NR) | | ***z*-score** for difference on SBQ between those who had a Positive/Very Positive overall attitude towards AA/NA (*M* = 23.7) and those that had a Negative/Very Negative attitude (*M* = 22.1):  *z* = -2.074, *p* = .038;  ***z*-score** for difference on SBQ between those who were Likely/Very Likely to recommend AA/NA (*M* = 23.5) and those that were Unlikely/Very Unlikely (*M* = 22.1):  *z* = -2.270, *p* = .023 |
| **Dermatis, et al. (2004)**; USA; group-administered paper survey;  *N* = 322: Residents at two Daytop inpatient treatment facilities were polled;  men (74%), women (26%); *M*age (SD) = 34.17 (7.4); Caucasian (19%), Hispanic (19%), Black (53%), Asian/Other (2%);  Catholic (41%), Protestant (22%), Muslim (8%), Jewish (2%), Other (22%), None (5%);  Alcohol & substance use | Attitude: **Preference for spirituality-based interventions being featured in Therapeutic Community treatment**  (1: *not at all*; 5: *very much*): 3.49 (1.36);  **Client belief that the program should feature the Twelve-Step program/approach (AA) more** (1: *not at all*; 5: *very much*): 2.52 (1.46)  **Clients’ perceptions regarding the benefit to recovery of each of the following TC program components** (1: *not at all*; 5: *very much*): 22.84 (4.7)  **Acceptance of Therapeutic Community principles** (1: *not at all*; 5: *very much*): 18.41 (4.7)  R/S: **Religious/Spiritual attendance as a child** (1: *Never*; 5: *More than once a week*): NR  **Current practice of meditation or prayer** (1: *Never*; 4: *Always*): 2.84 (1.1);  **Belief in a higher power** (1: *Never*; 4: *Always*): Always (78%), Not always (22%);  **Adapted Intrinsic/Extrinsic Scale for Spiritual Orientation** (Feagin, 1964; 1: *Strongly agree*; 5: *Strongly disagree*, or 1: *Never*; 5: *More than* *once a week*): 18.16 (6.7) | | **Correlation** between preference for spirituality-based interventions being featured in Therapeutic Community treatment and:  Spiritual orientation to life (*r* = -.35***),  Frequency of prayer (*r* = .36***);  ***t*-test** for differences in Preference for a type of intervention being featured in Therapeutic Community treatment by belief in a higher power:  Spiritually-based: *t*(317) = 4.6*** (more spiritual = more endorsement);  12-step approach: *t* = NR (ns) |
| **Dermatis, et al. (2010)**; USA; paper survey;  *N* = 222: Residents at two Daytop inpatient treatment facilities were polled;  men (88%), women (12%); *M*age (SD) = 34.9 (9.3); Caucasian (23%), Black (45%), Latino/Other (32%);  Catholic (45%), Protestant (30%), Muslim/Other (15%), None (10%);  Alcohol & substance use | Attitude: **Acceptance and perceived efficacy of Therapeutic Community principles**  (Metrikin, Galanter, Dermatis, & Bunt, 2003; 1: *not at all*; 5: *very much*): NR;  R/S: **Adapted Intrinsic/Extrinsic Scale for Spiritual Orientation** (Feagin, 1964; 1: *Strongly agree*; 5: *Strongly disagree*, or 1: *Never*; 5: *More than* *once a week*): NR;  **Current practice of meditation or prayer** (1: *Never*; 4: *Always*): NR;  **Belief in a higher power** (1: *Never*; 4: *Always*): NR;  **Feeling connected to others** (1: *Never*; 4: *Always*): NR | | **Correlation** between Spiritual Orientation and Acceptance of Therapeutic Community principles: *r* = .233***  **Regression** predicting acceptance of therapeutic community principles, after controlling for age, gender, gang membership, 12-step group attendance, and cannabis drug problem:  Spiritual Orientation: 𝛽 = .166*** |
| **Diallo (2013)**; USA; paper survey;  *N* = 84: Clients recruited from four addiction treatment facilities;  *M*age (SD) = 43.84 (11.11); Caucasian (70.5%), Black (22.19%), Hispanic (4.8%), Asian (2%);  Christian (80%), Atheist (11%), Muslim (1%), None (10%);  NR | Attitude: **Willingness for counsellor to include religion/spirituality in sessions** (% that agreed):  In general (97.6%),  If the counsellor is knowledgeable about their r/s but has a different personal r/s (63.1%),  If the counsellor is not knowledgeable about their r/s but has the same r/s (26.2%);  **Preference of counsellor’s knowledge vs experience about their religion/spirituality** (*n* = 80; 1: *counsellor with the same religion/spirituality as them but not informed/ knowledgeable about their religion or spirituality*; 2: *counsellor with a different religion from theirs but informed/knowledgeable about their religion/ spirituality*; 3: *neither*; % that agreed):  1 = 13.1%, 2 = 59.5%, 3 = 21.4%;  R/S: **Religious Affiliation** | | **Logistic regression** analysis predicting willingness for counsellor to include religion/spirituality in sessions, including age, gender, and education:  Religious affiliation:  In general (OR = 10.74*; 95% CI = [1.22, 94.14]),  If the counsellor is knowledgeable about their r/s but has a different personal r/s (OR = 5.738 (ns); 95% CI = [.639, 51.550]),  If the counsellor is not knowledgeable about their r/s but has the same r/s (OR = 0.33 (ns); 95% CI = [0.03, 3.0]) |
| **Droubay & Butters (2020)**; USA; online survey;  *N* = 136: Social work University students recruited via classes;  men (25.7%), women (74.3%); Aged 18-24 (21%), Aged 25-34 (46%), Aged 35-44 (20%), Aged 45+ (14%); Caucasian (83.8%), Non-White (16.2%): Hispanic/Latino (8%); Heterosexual (86.8%), LGBTQ+ (13.2%): Bisexual (7%), Gay or Lesbian (4%); Political affiliation (0: *Conservative*; 4: *Liberal*) = 2.6 (.795);  Catholic (5%), Latter-day Saints (38%), Religious “none” including atheist, agnostic (46%)  Pornography | Attitude: **Perceived addictiveness of pornography** (0: *not at all*; 4: *very much*): 2.680 (1.173);  **Perception of pornography as a public health issue** (0: *not at all serious*; 4: *very serious*): 2.000 (1.355);  R/S: **Santa Clara Strength of Religious Faith Questionnaire** (Plante & Boccaccini, 1997; 1: *Strongly disagree*; 10: *Strongly agree*, or 1: Never; 5: *More than once a week*; range = 0-30):  15.130 (10.665) | | **Mediation analysis** predicting the view of pornography as a public health issue, with perceived addictiveness of pornography as the mediator (Coefficient (SE)):  *a:* Religiosity predicting addictiveness of pornography = .046 (.010)***;  *b*: Addictiveness of pornography predicting pornography as a public health issue = .628 (.077)***;  *c:* Religiosity predicting pornography as a health issue = .046 (NR)***;  *c’:* Religiosity predicting pornography as a health issue mediated by pornography’s addictiveness = .016 (.009), *p* = .083, standardized indirect effect: point estimate = .224, 95% BCa CI = .120 to .325 |
| **Edelstein, et al. (2020)**; Israel; questionnaire;  *N* = 540: Students from the nursing (*n* = 35.2%), social work (*n* = 34.6%), and medical (*n* = 30.2%) departments volunteered;  men (19.6%), women (80.4%); *Median*Age = 25 (IQ range = 24-27); Jewish (91.9%); Bedouin-Arab (8.1%);  Religious (30.6%), Secular (69.4%);  Cannabis | Attitude: **Agreement with each Cannabis Belief:**  Physicians should recommend cannabis as a medical therapy (92.2%);  There are significant physical and mental health benefits using medical cannabis (96.1%);  Cannabis should be legalized for recreational use (60.9%);  Cannabis can be addictive (reported = 86.8%, calculated = 86.1%);  Using cannabis poses serious physical health risks (52.4%);  Using cannabis poses serious mental health risks (64.4%);  R/S: **Religious Affiliation** | | **Chi-squared and Fisher’s exact Test** for Agreement with each Cannabis Belief between religious and secular participants (religious vs secular):  Physicians should recommend cannabis as a medical therapy (89 vs 94.9%*, *p* = .013);  There are significant physical and mental health benefits using medical cannabis (95.1 vs 97.1% (ns), *p* = .265);  Cannabis should be legalized for recreational use (30.4 vs 70%***, *p* < .001);  Cannabis can be addictive (89.7 vs 85.4% (ns), *p* = .180);  Using cannabis poses serious physical health risks (63 vs 48.1%***, *p* = .001);  Using cannabis poses serious mental health risks (72.1 vs 61.6%*, *p* = .018) |
| **Flórez, et al. (2015)**; USA; survey;  *N* = 1,235: Church attendees at churches: two African American Baptist, two Latino Pentecostal, and one large Latino Roman Catholic;  men (36.8%), women (63.2%); Aged 18-30 (29.2%), Aged 31-40 (45.6%), Aged 51+ (25.2%); African American (34.4%), Latino (65.6%);  Christian (100%);  Drugs | Attitude: **Modified Alcoholism Stigma Scale** (Ronzani, Higgins-Biddle, & Furtado, 2009; 1: *Strongly disagree*; 5: *Strongly agree*): 3.2 (1.3);  R/S: **Religious Affiliation**;  **Church Attendance**: NR | | **Mean score** for drug addiction stigma within those who attend church: 3.2 (1.3) |
| **Globetti, Alsikafi, & Christy (1977)**; U.S. military community in Europe; structured questionnaire interview;  *N* = 230: Students in American military schools were recruited;  men (40.9%), women (58.3%); Aged 15 (20.4%), Aged 16 (35.2%), Aged 17 (30.9%), Aged 18 (10%); White (76.1%), Black (18.7%);  Affiliation (NR);  Alcohol | Attitude: **Attitude towards excessive drinking** (Alcohol abuse; 1: *Strongly agree*; 5: *Strongly agree*): Permissive (1-2.5) = 20%, Ambivalent (2.51-3) = 33%, Non-permissive (3.01-5) = 46%;  R/S: **Religious Attendance**: Once a week (32.6%), Once a month (13.5%), Twice a year (8.7%), Rarely (39.1%) | | **Chi-squared test** between attitudes and religious attendance: 𝜒^2^ = 15.38**:  Once a week (*n* = 75): Permissive (16%), Ambivalent (25%), Non-permissive (59%);  Once a month (*n* = 31): Permissive (23%), Ambivalent (52%), Non-permissive (26%);  Twice a year (*n* = 20): Permissive (10%), Ambivalent (40%), Non-permissive (50%);  Rarely (*n* = 90): Permissive (26%), Ambivalent (37%), Non-permissive (37%) |
| **Goldfarb, et al. (1996);** USA; questionnaire;  *N* = 220: Inpatient dually-diagnosed patients and medical students recruited from a hospital;  Patients (*n* = 101): men (76%), women (24%); *M*Age (SD) = 36.7 (8.6); White (21.8%), African-American (45.5%); Hispanic (28.7%), Other (3%);  Roman Catholic (41.6%), Protestant (41.6%), Jewish (2%), Muslim (3%), Other (12%);  Student (*n* = 119): men (61%), women (39%); *M*Age (SD) = 23.5 (2); White (52.9%), African-American (3.4%); Hispanic (3.4%), Asian (32.8%), Other (7.6%);  Roman Catholic (21.8%), Protestant (13.4%), Jewish (31.9%), Muslim (3.4%), Hindu (3.4%), Other (26.1%);  Substance use | Attitude: **Perception of the importance of aspects of treatment for dually diagnosed** (1: *Not at all*; 5: *Very much*; and rank ordered):  Housing: Students = 4.57 (.68): 74% ranked at 1, 2, or 3,  Patients = 4.57 (.92): 43% ranked at 1, 2, or 3,  Students’ perception of patients = 4.35 (.83);  Government Benefits: Students = 4.43 (.88): 64% ranked as 1, 2, or 3,  Patients = 4.06 (1.27): 19% ranked as 1, 2, or 3,  Students’ perception of patients = 3.36 (.93);  A sense of inner peace: Students = 3.31 (1.07),  Patients = 4.66 (.84),  Students’ perception of patients = 4.02 (.99);  Medical services: Students = 4.06 (1.02),  Patients = 4.65 (5.08),  Students’ perception of patients = 4.23 (.85);  A Job: Students = 3.97 (1.15),  Patients = 3.71 (1.56),  Students’ perception of patients = 4.23 (.92);  Belief in God/Higher Power: Students = 3.15 (.94): 10% ranked as 1, 2, or 3 and 39% ranked as 9, 10, or 11,  Patients = 4.65 (.91): 59% ranked as 1, 2, or 3,  Students’ perception of patients = 2.82 (1.14);  Outpatient treatment: Students = 3.99 (.92),  Patients = 4.29 (1.02),  Students’ perception of patients = 4.30 (.67);  AA meetings: Students = 2.86 (1.13): 7% ranked as 1, 2, or 3,  Patients = 4.61 (.80): 50% ranked as 1, 2, or 3,  Students’ perception of patients = 4.10 (.85);  Strong spiritual orientation: Students = 2.95 (.92),  Patients = 4.48 (.93),  Students’ perception of patients = 3.03 (1.10);  Supportive community/place of worship: Students = 3.65 (.95),  Patients = 4.09 (1.29),  Students’ perception of patients = 3.95 (.95);  Trusting people: Students = 3.32 (1.07),  Patients = 3.93 (1.37),  Students’ perception of patients = 4.14 (.81);  **Perception of improving each aspect of treatment for recovery** (1: *Not at all*; 5: *Very much*; and rank ordered):  More groups on spirituality: Students = 3.03 (1.05): 19% ranked 1 or 2,  Patients = 3.86 (1.34): 60% ranked as 1 or 2,  Students’ perception of patients = 2.73 (.95);  More access to religious services: Students = 2.87 (1.08),  Patients = 3.78 (1.46),  Students’ perception of patients = 2.74 (.99)  Better food: Students = 4.16 (.93): 62% ranked as 1 or 2,  Patients = 3.53 (1.50): 23% ranked as 1 or 2,  Students’ perception of patients = 3.43 (1.09)  Nicer hospital rooms: Students = 4.07 (.91),  Patients = 2.95 (1.53),  Students’ perception of patients = 2.82 (1.06)  More contact with doctors: Students = 3.86 (.99),  Patients = 4.45 (.96),  Students’ perception of patients = 3.58 (1.01)  More movies/entertainment: Students = 3.92 (1.01),  Patients = 2.50 (1.50),  Students’ perception of patients = 2.72 (1.26);  R/S: **Adapted Intrinsic/Extrinsic Scale for Spiritual Orientation** (Feagin, 1964; 1: *Strongly agree*; 5: *Strongly disagree*, or 1: *Never*; 5: *More than* *once a week*): Patients = 16.7 (6.49); Students = 23.24 (6.6)  **Gallup poll report on Religion in America (1990):**  Belief in God: Students (72.6%), Patients (96.7%)  God or a Universal Spirit is a heavenly father who can be reached by prayer: Students (26.1%), Patients (75.6%)  God/Universal Spirit is an idea not a being: Students (19.3%), Patients (6.7%);  God/Universal Spirit is an impersonal creator: Students (5%), Patients (2.2%);  Don’t Know what God/Universal Creator is: Students (33.6%), Patients (12.2%) | | ***t*-test** patients were seen to be more spiritual than students: *t* = 8.7***;  ***t*-test** between perceptions of the importance of each aspect of treatment in students vs patients, students’ perception of patients vs patients, & students vs students’ perceptions of patients:  Housing: .03 (ns), 1.88 (ns), 2.32*;  Government benefits: 2.55***, 4.73***, 9.6***;  Inner peace: 10.28***, 5.17***, 5.96*;  Medical services: 5.08***, 4.15***, 1.56 (ns);  A job: 1.43 (ns), 3.13*, 2.12*;  Belief in God/Higher Power: 12.04***, 13***, 2.4*;  Outpatient treatment: 2.26*, .13 (ns), 3.26*;  AA meetings: 13.09***, 4.59***, 9.80***;  Strong spiritual orientation: 12.18***, 10.40***, .68 (ns);  Supportive community: 2.92*, .99 (ns), 2.68*;  Trusting people: 3.72***, 1.42 (ns), 7.68***;  ***t*-test** between perceptions of the importance of improving each aspect of treatment in students vs patients, students’ perception of patients vs patients, students vs students’ perceptions of patients:  More groups on spirituality: 5.13***, 7.23***, 2.5*;  More access to religious services: 5.3***, 6.22***, .92 (ns);  Better food: 3.78***, .61 (ns), .34***;  Nicer hospital rooms: 6.69***, .72 (ns), 10.87***;  More contact with doctors: 4.44***, 6.46***, 2.12*;  More movies/entertainment: 8.38***, 1.23 (ns), 8.44*** |
| **Gritsenko, et al. (2020)**; Russia; paper questionnaire;  *N* = 463: Medical students were given the option to complete the questionnaire during a class;  men (55%), women (45%); *Median*Age (SD) = 20 (NR), First year (16.4%), Second year (35.4%), Third year (33.3%), Fourth year (11.4%), Fifth year (2.6%), Sixth year (.9%);  Christian (58.4%), Muslim (28.4%), Jewish (.4%), Non-denomination (12.8%);  Cannabis | Attitude: **Beliefs about cannabis (per RADAR research group)**:  Would recommend medical cannabis for patient use (32.6%),  There are significant physical health benefits using medical cannabis (32.6%),  There are significant mental health benefits using medical cannabis (34.5%),  Cannabis should be legalized for recreational use (24%),  Cannabis is not addictive (22.5%),  Using cannabis does not pose serious physical health risks (29.8%),  Using cannabis does not pose serious mental health risks (26.1%),  Additional research regarding medical cannabis use should be encouraged (62.2%);  R/S: **Religiosity**: Religious (79.5%), Secular (20.5%) | | **Chi-squared test** for differences between religiosity groups for each belief about cannabis (𝜒^2^):  Would recommend medical cannabis for patient use = NR***,  There are significant physical health benefits using medical cannabis = NR***,  There are significant mental health benefits using medical cannabis = NR***,  Cannabis should be legalized for recreational use = NR, *p* = .003,  Cannabis is not addictive = NR, *p* = .593,  Using cannabis does not pose serious physical health risks = NR, *p* = .02,  Using cannabis does not pose serious mental health risks = NR, *p* = .024,  Additional research regarding medical cannabis use should be encouraged = NR, *p* = .009 |
| **Gurel & Spain (1977)**; USA; questionnaire;  *N* = 53: Nurses with no prior alcohol or substance use education were recruited;  Comfort in providing care to patients with alcohol problems: Comfortable Catholic (32.1%), Comfortable Non-sectarian (22.6%), Uncomfortable Catholic (13.2%), Uncomfortable Non-sectarian (32.1%);  Catholic (45.3%), Non-sectarian (54.7%);  Alcohol | Attitude: **Marcus Alcoholism Questionnaire** (Marcus, 1980; 1: *agree*; 7: *disagree*):  Emotional difficulties (Factor 1): Comfortable Catholic = 5.06 (.93), Comfortable Non-sectarian = 4.95 (.8), Uncomfortable Catholic = 4.6 (.98), Uncomfortable Non-sectarian = 5.22 (.93);  Loss of control (Factor 2): Comfortable Catholic = 4.25 (.92), Comfortable Non-sectarian = 4.55 (.89), Uncomfortable Catholic = 3.95 (1.80), Uncomfortable Non-sectarian = 4.88 (.97);  Prognosis for recovery (Factor 3): Comfortable Catholic = 1.88 (.64), Comfortable Non-sectarian = 1.98 (.69), Uncomfortable Catholic = 2.4 (.98), Uncomfortable Non-sectarian = 1.98 (.68);  Steady drinking (Factor 4): Comfortable Catholic = 5.38 (.97), Comfortable Non-sectarian = 5.73 (1.0), Uncomfortable Catholic = 5.25 (1.19), Uncomfortable Non-sectarian = 5.58 (.88);  Character defect (Factor 5): Comfortable Catholic = 2.10 (.99), Comfortable Non-sectarian = 2.59 (.86), Uncomfortable Catholic = 2.85 (.75), Uncomfortable Non-sectarian = 2.73 (.98);  Social status (Factor 6): Comfortable Catholic = 2.26 (.91), Comfortable Non-sectarian = 2.11 (.71), Uncomfortable Catholic = 2.3 (.74), Uncomfortable Non-sectarian = 2.18 (.73);  Illness conception (Factor 7): Comfortable Catholic = 1.96 (1.06), Comfortable Non-sectarian = 2.02 (.75), Uncomfortable Catholic = 2.25 (.88), Uncomfortable Non-sectarian = 2.20 (.63);  Harmless indulgence (Factor 8): Comfortable Catholic = 1.83 (.58), Comfortable Non-sectarian = 2.34 (1.01), Uncomfortable Catholic = 2.35 (.86), Uncomfortable Non-sectarian = 2.17 (.74);  Addiction liability (Factor 9): Comfortable Catholic = 5.13 (.88), Comfortable Non-sectarian = 5.05 (.67), Uncomfortable Catholic = 5.4 (.45), Uncomfortable Non-sectarian = 4.87 (.86);  R/S: **Religious affiliation** | | **Sign test**: NR: Uncomfortable Catholic had less favourable attitudes than all other groups on factors 1-8 (*p* < .02) |
| **Hatchett, et al. (2011)**; USA; mailed survey;  *N* = 94: Clergy at churches on the Texas/Mexico border were mailed surveys via targeted sampling;  men (91.3%), women (6.7%); *M*Age = 47 (NR); Caucasian (71.3%), Hispanic (22.3%), African American (4.3%), Asian (2.1%);  Christian (100%);  Alcohol | Attitude: **Attitudes of Clergy about Alcohol Use** (Hatchett, 2002): ***Definitions of alcohol abuse***:  A reaction to life stress: Agree (69.2%), Disagree (30.8%);  A learned behaviour: Agree (84.8%), Disagree (15.2%);  A genetic problem: Agree (44.8%), Disagree (55.2%);  Having weak will power: Agree (39.1%), Disagree (60.9%);  A moral problem: Agree (75.6%), Disagree (24.4%);  Caused by peer pressure: Agree (65.5%), Disagree (34.5%);  ***Perceptions of importance of reasons for abstaining***: Health related issues: Very (73.4%), Somewhat (21.3%), Not (3.2%);  Fear of becoming alcoholic: Very (51.1%), Somewhat (30.9%), Not (14.9%);  Causing regretting behaviour that occurred while drinking: Very (69.1%), Somewhat (16%), Not (11.7%);  Cost issues: Very (47.9%), Somewhat (37.2%), Not (12.8%);  Upsetting family/friends: Very (48.9%), Somewhat (37.2%), Not (11.7%);  Potential work problems: Very (81.9%), Somewhat (11.7%), Not (4.3%);  Religious incompatibility: Very (51.1%), Somewhat (21.3%), Not (25.5%);  Causing sickness: Very (30.9%), Somewhat (43.6%), Not (22.3%);  Trouble with police: Very (59.6%), Somewhat (26.6%), Not (10.6%);  Loss of control over life: Very (89.4%), Somewhat (5.3%), Not (3.2%);  ***Attitudes about alcohol-related practices*** (*n* = 91): Some kinds of sickness are helped by alcohol: Agree (45.1%), Disagree (54.9%);  Where I grew up some people used alcohol as a medicine for physical problems: Agree (40.7%), Disagree (59.3%);  Alcohol can’t damage your body if you always drink in moderation: Agree (29.7%), Disagree (70.3%);  People should watch how much alcohol they have to avoid gaining weight (*n* = 90): Agree (32.2%), Disagree (67.8%);  Beer is less harmful to the body than other alcoholic drinks: Agree (9.9%), Disagree (90.1%);  Women who are drunk should always be punished (*n* = 88): Agree (21.6%), Disagree (78.4%);  Men who are drunk should always be punished (*n* = 88): Agree (21.6%), Disagree (78.4%);  There is nothing good to be said about drinking (*n* = 92): Agree (45.7%), Disagree (52.2%);  Getting drunk is just an innocent way of having fun (*n* = 92): Agree (1.1%), Disagree (98.9%);  Drinking is one of the main causes of people doing things they shouldn’t (*n* = 92): Agree (73.9%), Disagree (26.1%);  People would lose respect for a man who spends any time at bars (*n* = 92): Agree (48.9%), Disagree (51.1%);  People would lose respect for a woman who spends any time at bars (*n* = 92): Agree (58.7%), Disagree (41.3%);  If I had a problem with drinking I would be ashamed to tell anyone: Agree (36.3%), Disagree (63.7%);  If members of my church were drinking, I would not know where to send them for help: Agree (5.5%), Disagree (94.5%);  People who drink have more fun (*n* = 90): Agree (1.1%), Disagree (98.9%);  People who drink have more friends: Agree (3.3.%), Disagree (96.7%);  ***Getting drunk***: NR;  R/S: **Religious Affiliation** | **Chi-squared analyses** between agreement with alcohol use questions: ***Definitions of alcohol abuse***:  A reaction to life stress: χ^2^ = 15.38***;  A learned behaviour: χ^2^ = 44.52***;  A genetic problem: χ^2^ = .93 (ns);  Having weak will power: χ^2^ = 4.15*;  A moral problem: χ^2^ = 23.51***;  Caused by peer pressure: χ^2^ = 8.38**;  ***Perceptions of importance of reasons for abstaining***: Health related issues: χ^2^ = 76.59***;  Fear of becoming alcoholic: χ^2^ = 19.14***;  Causing regretting behaviour that occurred while drinking: χ^2^ = 59.69***;  Cost issues: χ^2^ = 18.67***;  Upsetting family/friends: χ^2^ = 20.89***;  Potential work problems: χ^2^ = 105.8***;  Religious incompatibility: χ^2^ = 14.96**;  Causing sickness: χ^2^ = 6.68*;  Trouble with police: χ^2^ = 36.29***;  Loss of control over life: χ^2^ = 139.2***;  ***Attitudes about alcohol-related practices*** (*n* = 91): Some kinds of sickness are helped by alcohol: χ^2^ = .89 (ns);  Where I grew up some people used alcohol as a medicine for physical problems: χ^2^ = 3.18 (ns);  Alcohol can’t damage your body if you always drink in moderation: χ^2^ = 15.04***;  People should watch how much alcohol they have to avoid gaining weight (*n* = 90): χ^2^ = 11.38**;  Beer is less harmful to the body than other alcoholic drinks: χ^2^ = 58.56***;  Women who are drunk should always be punished (*n* = 88): χ^2^ = 28.41***;  Men who are drunk should always be punished (*n* = 88): χ^2^ = 28.41***;  There is nothing good to be said about drinking (*n* = 92): χ^2^ = .43 (ns);  Getting drunk is just an innocent way of having fun (*n* = 92): χ^2^ = 88.04***;  Drinking is one of the main causes of people doing things they shouldn’t (*n* = 92): χ^2^ = 21.04***;  People would lose respect for a man who spends any time at bars (*n* = 92): χ^2^ = .04 (ns);  People would lose respect for a woman who spends any time at bars (*n* = 92): χ^2^ = 2.78 (ns);  If I had a problem with drinking I would be ashamed to tell anyone: χ^2^ = 6.87**;  If members of my church were drinking, I would not know where to send them for help: χ^2^ = 72.10***;  People who drink have more fun (*n* = 90): χ^2^ = 86.04***;  People who drink have more friends: χ^2^ = 79.40***;  ***Getting drunk*** was always deemed inappropriate regardless of situation, age, or gender | |
| **Hecker, et al. (1995)**; USA; Mailed survey and one out of four randomly selected vignettes;  *N* = 199: Randomly chosen sample (*n* = 400) of clinical members of the American Association of Marriage and Family Therapy;  men (43.7%), women (56.3%); *M*Age = 49.64 (8.86); Vignette 1 (Married monogamous female, *n* = NR), Vignette 2 (Married monogamous male, *n* = NR), Vignette 3 (Single female with varied partners, *n* = NR), Vignette 4 (Single male with varied partners, *n* = NR);  Protestant (49.2%), Catholic (16.1%), Jewish (14.6%), Mormon (2%), None (9%), Other (9%)  Sex | Attitude: **Level of perceived sexual pathology** (1: *None*; 5: *Extreme*): Time 1 = 74 (13.8), Time 2 = 62.8 (16.9);  **Perceived degree to which client is a “sex addict”** (1: *Definitely not*; 5: *Definitely yes*): Time 1 = 3.9 (2.6), Time 2 = 4.5 (3.2);  **Perceived number of sessions of therapy required** (1: *0-10*, 2: *11-20*, 3: *21-30*, 4: *31-40*, 5: *40+*):  **Perceived best treatment** (1: N*o therapy*, 2: *Supportive/educative therapy*, 3: *short-term therapy*, 4: *long-term therapy*);  **Prediction of client’s outcome** (1: *Very positive*; 5: *Very negative*):  R/S: **Religiosity**: High (60.3%): Strongly (29.6%), Moderately (30.7%); Low (39.2%): Slightly (18.6%), Not (20.6%) | | **MANOVA** predicting all of the attitude outcomes:  Three-way interaction between Vignette, sex of respondent, and religiosity: *F*(15,432) = .694, *p* = .46: Univariate comparisons: sexual pathology *F* (3, 146) = .980 (ns), “sex addict” *F* (3, 146) = .895 (ns), sessions *F* (3, 146) = .689 (ns), best treatment *F* (3, 146) = .457 (ns), client outcome *F* (3, 146) = .779 (ns);  Two-way interaction between sex of respondent and religiosity: *F* (5, 142) = 2.144, *p* = .064: Univariate comparisons:  Sexual pathology *F* (1, 146) = 4.693, *p* = .032: Males with high religiosity (*M* = 3.031) > (Males with low religiosity (*M* = 2.381) ≅ Females with low religiosity (*M* = 2.296) ≅ Females with high religiosity (*M* = 2.333));  Sessions *F* (1, 146) = 3.939, *p* = .049: Males with low religiosity (*M* = 1.90) < Females with low religiosity (*M* = 2.353), Males with high religiosity (*M* = 2.609) > Females with high religiosity (*M* = 2.160)  Two-way interaction between Vignette and religiosity: *F* (15, 432) = 1.024, *p* = .429  Main effects for religiosity: “sex addict”: *F* (1, 156) = 5.152, *p* = .021: high religiosity (*M* = 2.551) > low religiosity (*M* = 2.483) |
| **Hshieh & Srebalus (1997)**; USA; Mailed questionnaire;  *N* = 250: Randomly selected psychologists affiliated with the American Psychological Association (APA) and alcoholism counsellors affiliated with the National Association of Alcoholism and Drug Abuse Counsellors;  Psychologists (*n* = 119): men (85%), women (15%);  Counsellors (*n* = 110): men (32%), women (68%);  Alcohol | Attitude: **Agree with disease model**: Psychologists: Yes (66%), No (34%); Counsellor: Yes (98%), No (2%);  **Treatment approach include 12-step model**: Psychologist: Yes (62%), No (38%); Counsellor: Yes (86%), No (14%);  **Favour abstinence as a treatment goal**: Psychologist: Yes (49%), No (51%); Counsellor: Yes (81%), No (19%);  **Believe controlled drinking and abstinence can be effective**: Psychologist: Yes (45%); Counsellor: Yes (17%);  R/S: **Religiousness**: Spiritual/Religious/Both: Psychologist (83%); Counsellor (95%) | | **Chi-squared test** between those who identified as religious/spiritual and those that did not (𝜒^2^):  Belief in disease concept = 18.47*** (50% of those who did not identify as r/s agreed),  12-step model = 14.46** (44% of those who did not identify as r/s agreed) |
| **Jolly & Orford (1983)**; UK; One of two differently titled questionnaires: Problem (*n* = 37) vs Normal (*n* = 39) behavior focused;  *N* = 76, 12 extra students were also included in the final analyses only: University students were approached randomly at the Christian Union and University living areas;  Christian Union (*n* = 39 + 5 extra): men (85%), women (15%); Problem questionnaire (*n* = 17), Normal questionnaire (*n* = 22);  Non-Christian Union (*n* = 37 + 7 extra): men (32%), women (68%); Problem questionnaire (*n* = 20), Normal questionnaire (*n* = 17)  Alcohol | Attitude: **Attitudes towards social drinking** (Strassburger & Strassburger, 1965; -3: *Strongly disagree*; 3: *Strongly agree*; range = -27-27):  Christian Union: Problem questionnaire = -1.94 (7.65),  Normal questionnaire = -2.22 (8.08);  Non-Christian Union: Problem questionnaire = 6.9 (7.56),  Normal questionnaire = 8.82 (6.85)  **Attitudes towards alcoholism and the alcoholic** (Strassburger & Strassburger, 1965; -3: *Strongly disagree*; 3: *Strongly agree*; range = -27-27):  Christian Union: Problem questionnaire = 8.11 (6.07),  Normal questionnaire = 5.68 (5.06);  Non-Christian Union: Problem questionnaire = 10 (4.97),  Normal questionnaire = 12.05 (6.47)  R/S: **Religious observance** (adapted from O’Connor, 1978; range = 2-43):  Christian Union: Problem questionnaire = 40.23 (4.07), Normal questionnaire = 40.4 (2.23);  Non-Christian Union: Problem questionnaire = 16.2 (12.68), Normal questionnaire = 14.82 (10.11) | | **ANOVA** including sex, questionnaire type, year of study in predicting attitudes: Christian Union vs Non-Christian Union:  Social drinking (*F* = 24.2***),  Alcoholism (*F* = 10.7**);  Non-Christian Union religious observance scores split into high and low by the median: ***t*-test** between high and low group’s attitude scores for:  Problem questionnaire:  Social drinking = 1.96 (ns),  Alcoholism = 1.92 (ns);  Normal questionnaire:  Social drinking = NR (ns),  Alcoholism = NR (ns) |
| **Lawrence, et al. (2012)**; USA; mailed survey;  *N* = 1,208: Stratified random sampling of psychiatrists and physicians;  *M*age (SD) = 45 (10.4);  Physicians (*n* = 896): men (64%), women (36%); Aged 25-36 (25%), Aged 37-44 (25%), Aged 45-53 (25%), Aged 54-65 (25%); White (71%), Black (6%), Asian (16%), Hispanic/Latino (5%), Other (2%);  Non-evangelical protestant (26%), Evangelical protestant (11%), Catholic/Orthodox (24%), Muslim (7%), Jewish (11%), Hindu (5%), Other (4%), No affiliation (11%);  Psychiatrists (*n* = 312): men (57%), women (43%); Aged 25-36 (26%), Aged 37-44 (13%), Aged 45-53 (29%), Aged 54-65 (31%); White (64%), Black (7%), Asian (21%), Hispanic/Latino (5%), Other (3%);  Non-evangelical protestant (23%), Evangelical protestant (7%), Catholic/Orthodox (22%), Muslim (3%), Jewish (13%), Hindu (8%), Other (9%), No affiliation (16%);  Alcohol | Attitude: **Effectiveness of** (For regression: Very effective: *very effective, somewhat effective*; Less effective: *not very effective, not at all effective*):  Participation in local AA: Physician: Very Effective (57%), Somewhat (42%), Not very (2%), Not (0%);  Psychiatrist: Very (64%), Somewhat (35%), Not very (1%), Not (0%);  Pharmacological therapy with addiction specialist physician: Physician: Very Effective (22%), Somewhat (62%), Not very (15%), Not (1%);  Psychiatrist: Very (31%), Somewhat (53%), Not very (15%), Not (1%);  Residential rehabilitation program:  Physician: Very Effective (38%), Somewhat (55%), Not very (7%), Not (0%);  Psychiatrist: Very (47%), Somewhat (50%), Not very (3%), Not (0%);  **Likelihood of referral to faith-based program** (For regression: Very likely: *very, somewhat*; Not very likely: *not very, not at all*):  Physician: Very (40%), Somewhat (39%), Not very (17%), Not (4%);  Psychiatrist: Very (29%), Somewhat (42%), Not very (22%), Not (7%)  **Agreement that an emphasis on spirituality is critical to 12-step program success**:  Physician: Strongly agree (39%), Somewhat agree (46%), Somewhat disagree (11%), Strongly disagree (4%);  Psychiatrist: Strongly agree (44%), Somewhat agree (37%), Somewhat disagree (14%), Strongly disagree (4%);  R/S: **Presented with a vignette of a Christian church-goer vs non-church goer** who has Alcohol use problems prior to attitudes questions;  **Importance of religion**: Physician: Not important/Not applicable (25%), Fairly (32%), Very (29%), Most (15%);  Psychiatrist: Not important/Not applicable (32%), Fairly (34%), Very (26%), Most (8%);  **Attendance at religious services**:  Physician: Never (14%), Once a month (48%), Twice a month (39%);  Psychiatrist: Never (17%), Once a month (52%), Twice a month (30%)  **Religious Affiliation** | | **Chi-squared** analysis between those given the church-goer vs non-church goer vignette:  All attitude measures NR (ns, *p* ≥ .09);  **Multiple logistic regression** for r/s predicting attitudes after adjusting for sex, race, age, and region of USA:  Participation in AA: Physician:  Affiliation (*p* = .07, ns): Non-Evangelical Protestant (53%): ≅ Evangelical protestant (58%; OR = 1.3, CI = .8-2.2), ≅Catholic (59%; OR = 1.3, CI = .8-2), ≅Muslim (68%; OR = 2.3, CI = .95-5.7), ≅Jewish (48%; OR = .9, CI = .5-1.5), ≅Hindu (74%; OR = 2.7, CI = .5-2.4), ≅Other (55%; OR = 1.1, CI = .5-2.4), ≅None (48%; OR = .9, CI = .5-1.5);  Importance of religion (*p* = .12, ns): Very important (54%) ≅ Not important (60%; OR = 1.1, CI = .96-1.3);  Psychiatrist:  Affiliation* (*p* = .02): Non-Evangelical Protestant (76%): ≅Evangelical Protestant (65%; OR = .5, CI = .1-1.7), ≅Muslim (63%; OR = .3, CI = .1-1.3), > Jewish (44%; OR = .3*, CI = .1-1.7), > Catholic (57%; OR = .4*, CI = .2-.8), ≅Hindu (71%; OR = .3, CI = .1-1.3), ≅Other (81%; OR = 1, CI = .3-3.4), ≅None (65%; OR = .6, CI = .3-1.4);  Importance of religion (*p* = .6, ns): Very important (67%) ≅ Not important (63%; OR = 1, CI = .1-1.3);  Pharmacotherapy: Physician:  Affiliation: Non-Evangelical Protestant (16%): < Catholic (23%; OR = 1.9*, CI = 1.1-1.3), < Muslim (44%; OR = 4.6*, CI = 1.8-11.7), < Other (32%; OR = 2.7*, CI = 1.2-6.5);  Importance of religion = ns;  Psychiatrist: Affiliation = ns, Importance of religion = ns;  Residential rehabilitation: Physician:  Affiliation = ns,  Importance of religion = ns;  Psychiatrist: affiliation = ns,  Importance of religion = ns;  Likelihood of referral: Physician:  Affiliation***: Non-Evangelical Protestant (40%): < Evangelical Protestant (76%; OR = 5.1*, CI = 2.6-9.8), ≅Catholic (46%; OR = 1.4, CI = .9-2.3), ≅Muslim (35%; OR = .6, CI = .2-1.6), > Jewish (16%; OR = .4*, CI = .2-.8), ≅Hindu (33%; OR = .5, CI = .2-1.4), ≅Other (33%; OR = .7, CI = .3-1.8), > Unaffiliated (20%; OR = .4*, CI = .2-.7);  Importance of religion***: Very important (55%) > Not very important (28%; OR = 1.8*, CI = 1.5-2.1);  Psychiatrist: Affiliation***: Non-Evangelical Protestant (35%): ≅Evangelical Protestant (68%; OR = 3.6, CI = .9-14.3), ≅Catholic (27%; OR = .5, CI = .2-1.1), ≅Muslim (29%; OR = .3, CI = .04-2.2), ≅Jewish (16%; OR = .4, CI = .1-1.2), ≅Hindu (42%; OR = .3, CI = .1-1), > Other (19%; OR = .2*, CI = .1-.6), > Unaffiliated (16%; OR = .3*, CI = .1-.8);  Importance of religion***: Very important (48%) > Not very important (19%; OR = 1.9*, CI = 1.4-2.5);  Spirituality as critical: Physician:  Affiliation***: Non-Evangelical Protestant (90%): ≅Evangelical Protestant (97%; OR = 3.4, CI = .9-12), ≅Catholic (91%; OR = 1.3, CI = .6-2.5), ≅Muslim (92%; OR = 1.3, CI = .4-4.2), > Jewish (73%; OR = .4*, CI = .2-.7), ≅Hindu (84%; OR = .5, CI = .1-2.2), ≅Other (80%; OR = .5, CI = .2-1.3), > Unaffiliated (57%; OR = .1*, CI = .1-.3);  Importance of religion***: Very important (95%) > Not important (78%; OR = 2.3*, CI = 1.7-3);  Psychiatrist: Affiliation (*p* = .3, ns): Non-Evangelical Protestant (79%): ≅Evangelical Protestant (89%; OR = 2.4, CI = .5-12.3), ≅Catholic (90%; OR = 2.5, CI = .9-7), ≅Muslim (75%; OR = .6, CI = .1-3.5), ≅Jewish (83%; OR = 1.6, CI = .5-4.9), ≅ Hindu (87%; OR = 1.8, CI = .4-7.8), ≅Other (78%; OR = 1, CI = .3-3.4),≅None (71%; OR = .7, CI = .3-1.6);  Importance of religion***: Very important (94%) > Not Important (75%; OR = 2.3*, CI = 1.4-3.7) |
| **Linsky (1965)**; USA; Phone interview;  *N* = 305: Probability stratified sampling of the general population from Vancouver, Washington city phone directory by sex, marital status, and census tract;  *M*age (SD) = 33 (NR);  Catholic (12.5%), Protestant: Ecclesia (Episcopalian, Presbyterian, Congregationalist; 16.7%), Denominations (Lutheran, Methodist, Baptist, Disciples of Christ; 43.3%), Fundamentalists (Holiness, Pentecostal, Adventists; 4.3%), Did not respond (23.3%);  *n* = 120: Data from another paper using a sample of hospital staff from a Tuberculosis Hospital who have frequent contact with Alcoholism patients (Linsky, Fagan, & Heinemann, 1964);  Catholic (24.2%), Ecclesia (21.7%), Denominations (53.3%), Fundamentalists (7.5%);  Alcohol | Attitudes: **Adapted** **Attitudes towards moderate social drinking** (Pennington & Passey, 1960; range = 0-8):  General population: Catholic = 4.6, Ecclesia = 4.45, Denominations = 4.19, Fundamentalists = 3.23;  **Adapted Attitudes towards Alcoholics and Alcoholism** (Pennington & Passey, 1960; range = 0-8):  General population: Catholic = 4.67, Ecclesia = 5.26, Denominations = 4.53, Fundamentalists = 4.23;  Hospital Staff: Catholic = 5.31, Ecclesia = 5.62, Denominations = 5.04, Fundamentalists = 4.91;  **Adapted Attitudes towards the Treatment of Alcoholism** (Pennington & Passey, 1960; range = 0-8):  General population: Catholic = 5.74, Ecclesia = 6.29, Denominations = 5.62, Fundamentalists = 5.96;  Hospital Staff: Catholic = 5.72, Ecclesia = 6.23, Denominations = 6.03, Fundamentalists = 5.78;  **Belief about cause of Alcoholism**: General population:  Social drinking and alcohol itself: Catholic (10.5%), Ecclesia (13.8%), Denominations (20.1%), Fundamentalists (23.1%);  Moral character of alcoholic: Catholic (2.6%), Ecclesia (5.5%), Denominations (15.5%), Fundamentalists (7.6%);  **Belief about best treatment** (*n* = 201): General population: Medical treatment (35.8%), Psychological help (34.8%), Use of willpower (15.4%), Religious help (13.9%)  R/S: **Religious affiliation** | | ***F*-test** between R/S groups for attitudes towards:  General population (*df* = 3,230):  Moderate Social Drinking = 3.48* (Catholics> Ecclesia > Denominations > Fundamentalists),  Alcoholics and Alcoholism = 1.84 (ns; Ecclesia > (Catholic ≈ Denominations) > Fundamentalists),  Treatment of Alcoholism = 1.95 (ns; Ecclesia > (Catholic ≈ Denominations ≈ Fundamentalists));  Hospital Staff (*df* = 3,116): Alcoholics and Alcoholism = 2.968*, Treatment of Alcoholism = 1.273 (ns);  **Chi-squared analysis** between R/S group in General population sample for each of the best treatment options: 𝜒^2^ = 27.27**:  Catholic (*n* = 33): Medical Treatment (57.6%), Psychological help (24.2%), Use of Willpower (12.1%), Religious Help (6.1%);  Ecclesia (*n* = 51): Medical Treatment (41.2%), Psychological help (45.1%), Use of Willpower (3.9%), Religious Help (9.8%);  Denominations (*n* = 104): Medical Treatment (29.8%), Psychological help (34.6%), Use of Willpower (20.2%), Religious Help (15.4%);  Fundamentalists (*n* = 13): Medical Treatment (7.7%), Psychological help (23.1%), Use of Willpower (30.8%), Religious Help (38.5%) |
| **Loewenthal, et al. (2003)**; UK; paper/mailed questionnaire;  *N* = 161: Religious individuals were recruited using a one-in-five quasi-random selection from church and synagogue lists and via snowball sampling;  men (49.1%), women (50.9%); *M*age (SD) = 40.78 (15.75);  Jewish (43.5%); Protestant (56.5%)  Alcohol | Attitudes: **Biphasic Alcohol Effects Scale** (Martin, Earleywine, Musty, Perrine, & Swift, 1993; range = 0-70):  Stimulant effects: Jewish man = 26.71 (13.89), Jewish woman = 30.30 (15.18), Protestant man = 33.09 (14.45), Protestant woman = 33.41 (17.11);  Sedative effects: Jewish man = 29.72 (13.73), Jewish woman = 32.56 (14.77), Protestant man = 28.50 (16.12), Protestant woman = 30.08 (16.68)  **Perceptions of alcoholics and alcoholism** (Weiss & Moore, 1992):  Many alcoholics taper off and control drinking again:  Jewish (*n* = 68): Agree (5.9%), Don’t know (50%), Disagree (44.1%);  Protestant (*n* = 90): Agree (16.7%), Don’t know (33.3%), Disagree (50%);  Most alcoholics drink because they want to:  Jewish (*n* = 68): Agree (17.6%), Don’t know (45.6%), Disagree (36.8%);  Protestant (*n* = 88): Agree (39.8%), Don’t know (18.2%), Disagree (42%);  Alcoholics are morally weak individuals:  Jewish (*n* = 69): Agree (14.5%), Don’t know (37.7%), Disagree (47.8%);  Protestant (*n* = 90): Agree (7.8%), Don’t know (20%), Disagree (72.2%);  Alcoholism is an illness:  Jewish (*n* = 69): Agree (68.1%), Don’t know (20.3%), Disagree (11.6%);  Protestant (*n* = 90): Agree (70%), Don’t know (18.9%), Disagree (11.1%);  To recover, alcoholics have to quit forever:  Jewish (*n* = 69): Agree (58%), Don’t know (30.4%), Disagree (11.6%);  Protestant (*n* = 89): Agree (55.1%), Don’t know (28.1%), Disagree (16.9%);  **Attitudes towards alcohol usage** (drinking to cheer up when low, drinking to relax when stressed, drinking on social occasions, going to the pub, and being slightly intoxicated; regarding others and self; 0: *no*, 1: *sometimes*, 2: *yes*; range = 0-30):  All items: Jewish man = 20.17 (5.35), Jewish woman = 18.66 (5.93), Protestant man = 24.47 (4.81), Protestant woman = 22.74 (6.12);  If these reasons could happen for themselves and belief they would drink for this reason (self-items): Jewish man = 6.04 (2.24), Jewish woman = 5.25 (2.58), Protestant man = 7.89 (2.05), Protestant woman = 6.77 (2.79)  R/S: **Religiosity** (Loewenthal, MacLeod, & Cinnirella, 2002; engagement in religious behaviour; 0: *never*; 4: *daily*; range = 0-12): NR;  **Religious affiliation** | | **ANOVA** for attitudes, with age, depression, anxiety, and religiosity as covariates, including gender and (*F*):  Affiliation: Biphasic Alcohol Effects Scale:  Stimulant effects = 5.29*: Protestant > Jewish*;  Sedative effects = NR (ns);  Attitudes to alcohol use: all items = 29.25***: Jewish < Protestant*;  self-items = 20.53***: Jewish women < Protestant men and women*, Jewish men < Protestant men*, Jewish men ≅ Protestant women;  **Chi-squared** between Jewish and Protestant groups for response to perceptions of alcoholics and alcoholism (𝜒^2^):  Many alcoholics taper off and control their drinking again = 6.69*: Jewish < Protestant;  Most alcoholics drink because they want to = 16.07***: Jewish > Protestant (in text, reported as Protestant > Jewish);  Alcoholics are morally weak individuals = 9.83**: Jewish < Protestant (Protestant men > protestant women: Chi-squared = 7.4*);  Alcoholism is an illness = .7 (ns);  To recover, alcoholics have to quit forever = .87 (ns) |
| **Lucchetti, et al. (2014)**; Brazil; in-person questionnaire;  *N* = 3,007: Door-to-door, nationwide, multistage survey recruited participants at home;  Adults over 18 (*n* = 2,346), Aged 14-17 (*n* = 661); men (42.7%), women (57.3%); *M*age (SD) = 35.71 (17.91); White (48.8%), Black (10.8%), Brown (37.6%), Other (2.8%);  Catholic (67.3%), Evangelical Protestant (23.3%), Other (3.6%), None (5.8%)  Alcohol | Attitude: **Attitudes toward** (Y: *Increase/Yes/ Agree*; N: *Decrease/Same/ No/Disagree*):  Taxes on alcoholic beverages: Religiousness: Very important (*n* = 2,374): Y (59.4%), N (40.6%); Somewhat/Not important (*n* = 491): Y (47.3%), N (52.7%);  Religious Attendance: 1+ times/week (*n* = 1,582): Y (62.7%), N (37.3%); <1 times/week (*n* = 1,283): Y (50.6%), N (49.4%);  Religiously affiliated: Yes (*n* = 2,700): Y (58.5%), N (41.5%); No (*n* = 165): Y (37.6%), N (62.4%);  Protestant Denomination: Yes (*n* = 669): Y (70%), N (30%); Other/No (*n* = 2,031): Y (54.7%), N (45.3%);  Minimum legal age for sale of alcohol: Religiousness: Very important (*n* = 1,609): Y (87.3%), N (12.7%); Somewhat/Not important (*n* = 1,354): Y (77.6%), N (22.4%);  Religious Attendance: 1+ times/week (*n* = 1,644): Y (58.4%), N (41.6%); <1 times/week (*n* = 1,319): Y (49.2%), N (50.8%);  Religiously affiliated: Yes (*n* = 2,792): Y (55.2%), N (44.8%); No (*n* = 171): Y (39.8%), N (60.2%);  Protestant Denomination: Yes (*n* = 690): Y (60.6%), N (39.4%); Other/No (*n* = 2,102): Y (53.4%), N (46.6%);  Government advertising campaigns: Religiousness: Very important (*n* = 652): Y (50.8%), N (49.2%); Somewhat/Not important (*n* = 150): Y (43.3%), N (56.7%);  Religious Attendance: 1+ times/week (*n* = 421): Y (49.6%), N (50.4%); <1 times/week (*n* = 381): Y (49.1%), N (50.9%);  Religiously affiliated: Yes (*n* = 750): Y (50.3%), N (49.7%); No (*n* = 52): Y (36.5%), N (63.5%);  Protestant Denomination: Yes (*n* = 156): Y (55.8%), N (44.2%); Other/No (*n* = 594): Y (48.8%), N (51.2%);  School-based prevention programs: Religiousness: Very important (*n* = 1,770): Y (65%), N (35%); Somewhat/Not important (*n* = 320): Y (63.8%), N (36.2%);  Religious Attendance: 1+ times/week (*n* = 1,178): Y (64.3%), N (35.7%); <1 times/week (*n* = 912): Y (65.6%), N (34.4%);  Religiously affiliated: Yes (*n* = 1,987): Y (65.1%), N (34.9%); No (*n* = 103): Y (60.2%), N (39.8%);  Protestant Denomination: Yes (*n* = 494): Y (63.2%), N (36.8%); Other/No (*n* = 1,493): Y (65.7%), N (34.3%);  Alcohol treatment programs: Religiousness: Very important (*n* = 2,494): Y (85.8%), N (14.4%); Somewhat/Not important (*n* = 513): Y (86.9%), N (13.1%);  Religious Attendance: 1+ times/week (*n* = 1,668): Y (85.4%), N (14.6%); <1 times/week (*n* = 1,339): Y (86.3%), N (13.7%);  Religiously affiliated: Yes (*n* = 2,833): Y (85.8%), N (14.2%); No (*n* = 174): Y (86.2%), N (13.8%);  Protestant Denomination: Yes (*n* = 702): Y (87%), N (13%); Other/No (*n* = 2,131): Y (85.4%), N (14.6%);  Restrictions on hours for alcohol sales: Religiousness: Very important (*n* = 2,463): Y (81.5%), N (18.5%); Somewhat/Not important (*n* = 510): Y (68%), N (32%);  Religious Attendance: 1+ times/week (*n* = 1,648): Y (83.1%), N (16.9%); <1 times/week (*n* = 1,325): Y (74.4%), N (25.6%);  Religiously affiliated: Yes (*n* = 2,803): Y (80.3%), N (19.7%); No (*n* = 170): Y (61.8%), N (38.2%);  Protestant Denomination: Yes (*n* = 692): Y (84.8%), N (15.2%); Other/No (*n* = 2,111): Y (78.8%), N (21.2%);  More efforts by businesses to stop serving alcohol to drunk customers: Religiousness: Very important (*n* = 2,476): Y (91.8%), N (8.2%); Somewhat/Not important (*n* = 506): Y (87%), N (13%);  Religious Attendance: 1+ times/week (*n* = 1,654): Y (93%), N (7%); <1 times/week (*n* = 1,328): Y (88.5%), N (11.5%);  Religiously affiliated: Yes (*n* = 2,810): Y (94.5%), N (8.8%); No (*n* = 172): Y (86.6%), N (13.4%);  Protestant Denomination: Yes (*n* = 694): Y (93.9%), N (6.1%); Other/No (*n* = 2,116): Y (90.4%), N (9.6%);  Ban sale of alcohol at bakeries, pastry shops, and grocery stores: Religiousness: Very important (*n* = 2,468): Y (78.6%), N (21.4%); Somewhat/Not important (*n* = 506): Y (63.8%), N (36.2%);  Religious Attendance: 1+ times/week (*n* = 1,652): Y (80.6%), N (19.4%); <1 times/week (*n* = 1,322): Y (70.4%), N (29.6%);  Religiously affiliated: Yes (*n* = 2,804): Y (77%), N (23%); No (*n* = 170): Y (61.8%), N (38.2%);  Protestant Denomination: Yes (*n* = 694): Y (83.1%), N (16.9%); Other/No (*n* = 2,110): Y (74.9%), N (25.1%);  Bottles and cans should have more warning messages about alcohol problems than “drink responsibly”: Religiousness: Very important (*n* = 2,474): Y (955%), N (4.5%); Somewhat/Not important (*n* = 508): Y (92.7%), N (7.3%);  Religious Attendance: 1+ times/week (*n* = 1,657): Y (95.9%), N (4.1%); <1 times/week (*n* = 1,325): Y (93.9%), N (6.1%);  Religiously affiliated: Yes (*n* = 2,812): Y (95.4%), N (4.6%); No (*n* = 170): Y (88.2%), N (11.8%);  Protestant Denomination: Yes (*n* = 697): Y (97.6%), N (2.4%); Other/No (*n* = 2,115): Y (94.7%), N (5.3%);  Alcohol advertising on television should be banned: Religiousness: Very important (*n* = 1,985): Y (69.1%), N (30.9%); Somewhat/Not important (*n* = 989): Y (55.1%), N (44.9%);  Religious Attendance: 1+ times/week (*n* = 1,646): Y (71.5%), N (28.5%); <1 times/week (*n* = 1,328): Y (60.6%), N (39.2%);  Religiously affiliated: Yes (*n* = 2,803): Y (67.4%), N (32.6%); No (*n* = 171): Y (56.7%), N (43.3%);  Protestant Denomination: Yes (*n* = 697): Y (74%), N (26%); Other/No (*n* = 2,106): Y (65.1%), N (34.9%);  Alcohol manufacturers should be banned from sponsoring sporting/culture events:  Religiousness: Very important (*n* = 1,623): Y (57.8%), N (42.2%); Somewhat/Not important (*n* = 1,293): Y (45.2%), N (54.8%);  Religious Attendance: 1+ times/week (*n* = 1,611): Y (60.9%), N (39.1%); <1 times/week (*n* = 1,305): Y (49.2%), N (50.8%);  Religiously affiliated: Yes (*n* = 2,743): Y (56.4%), N (43.6%); No (*n* = 171): Y (43.3%), N (56.7%);  Protestant Denomination: Yes (*n* = 685): Y (64.1%), N (35.9%); Other/No (*n* = 2,060): Y (53.9%), N (46.1%);  Alcohol advertisements should reserve a space for warning messages: Religiousness: Very important (*n* = 2,466): Y (94.9%), N (5.1%); Somewhat/Not important (*n* = 512): Y (93.6%), N (6.4%);  Religious Attendance: 1+ times/week (*n* = 1,652): Y (95.6%), N (4.4%); <1 times/week (*n* = 1,326): Y (93.5%), N (6.5%);  Religiously affiliated: Yes (*n* = 2,806): Y (94.8%), N (5.2%); No (*n* = 172): Y (92.4%), N (7.6%);  Protestant Denomination: Yes (*n* = 697): Y (96.6%), N (3.4%); Other/No (*n* = 2,109): Y (94.2%), N (5.8%);  Alcohol treatment programs should be free and compulsory at health centers, clinics, and general public hospitals: Religiousness: Very important (*n* = 2,480): Y (97%), N (3%); Somewhat/Not important (*n* = 509): Y (95.7%), N (4.3%);  Religious Attendance: 1+ times/week (*n* = 1,656): Y (97.4%), N (2.6%); <1 times/week (*n* = 1,333): Y (95.9%), N (4.1%);  Religiously affiliated: Yes (*n* = 2,816): Y (94.3%), N (3.1%); No (*n* = 173): Y (94.8%), N (5.2%);  Protestant Denomination: Yes (*n* = 699): Y (98.3%), N (1.7%); Other/No (*n* = 2,117): Y (96.4%), N (3.6%);  It is very easy for persons under 18 to buy alcohol, despite it being illegal:  Religiousness: Very important (*n* = 2,475): Y (94.5%), N (5.5%); Somewhat/Not important (*n* = 508): Y (92.3%), N (7.7%);  Religious Attendance: 1+ times/week (*n* = 1,652): Y (95.3%), N (4.7%); <1 times/week (*n* = 1,331): Y (92.6%), N (7.4%);  Religiously affiliated: Yes (*n* = 2,811): Y (94.1%), N (5.9%); No (*n* = 172): Y (93.6%), N (6.4%);  Protestant Denomination: Yes (*n* = 698): Y (95%), N (5%); Other/No (*n* = 2,113): Y (93.8%), N (6.2%);  Increase supervision of businesses that sell alcohol to prevent sale to minors:  Religiousness: Very important (*n* = 2,473): Y (96.3%), N (3.7%); Somewhat/Not important (*n* = 508): Y (93.5%), N (6.5%);  Religious Attendance: 1+ times/week (*n* = 1,653): Y (97.3%), N (2.7%); <1 times/week (*n* = 1,328): Y (94.1%), N (5.9%);  Religiously affiliated: Yes (*n* = 2,811): Y (96.2%), N (3.8%); No (*n* = 170): Y (90.6%), N (9.4%);  Protestant Denomination: Yes (*n* = 698): Y (97.6%), N (2.4%); Other/No (*n* = 2,113): Y (95.7%), N (4.3%);  Others drinking and driving is a threat to my personal safety and the security of my family: Religiousness: Very important (*n* = 2,486): Y (97.8%), N (2.2%); Somewhat/Not important (*n* = 513): Y (96.9%), N (3.1%);  Religious Attendance: 1+ times/week (*n* = 1,662): Y (87%), N (3%); <1 times/week (*n* = 1,337): Y (98.4%), N (1.6%);  Religiously affiliated: Yes (*n* = 2,825): Y (97.7%), N (2.3%); No (*n* = 174): Y (96.6%), N (3.4%);  Protestant Denomination: Yes (*n* = 699): Y (97.4%), N (2.6%); Other/No (*n* = 2,126): Y (97.8%), N (2.2%);  Most people who drink and drive are alcoholics or problem drinkers: Religiousness: Very important (*n* = 2,466): Y (86.6%), N (13.4%); Somewhat/Not important (*n* = 512): Y (77.7%), N (22.3%);  Religious Attendance: 1+ times/week (*n* = 1,647): Y (87.2%), N (12.8%); <1 times/week (*n* = 1,331): Y (82.3%), N (17.7%);  Religiously affiliated: Yes (*n* = 2,805): Y (85.5%), N (14.5%); No (*n* = 173): Y (78.6%), N (21.4%);  Protestant Denomination: Yes (*n* = 696): Y (89.4%), N (10.6%); Other/No (*n* = 2,109): Y (84.2%), N (15.8%);  No problem driving when beginning to feel the effects of alcohol: Religiousness: Very important (*n* = 697): Y (81.6%), N (18.4%); Somewhat/Not important (*n* = 2,287): Y (83.3%), N (16.7%);  Religious Attendance: 1+ times/week (*n* = 1,653): Y (21.8%), N (78.2%); <1 times/week (*n* = 1,331): Y (25.2%), N (74.8%);  Religiously affiliated: Yes (*n* = 2,812): Y (23.2%), N (76.8%); No (*n* = 172): Y (26.7%), N (73.3%);  Protestant Denomination: Yes (*n* = 699): Y (19.9%), N (80.1%); Other/No (*n* = 2,113): Y (24.2%), N (75.8%);  A person drinking and driving will almost certainly be pulled over and detained:  Religiousness: Very important (*n* = 2,454): Y (64.5%), N (35.5%); Somewhat/Not important (*n* = 503): Y (57.5%), N (42.5%);  Religious Attendance: 1+ times/week (*n* = 1,643): Y (63.7%), N (36.6%); <1 times/week (*n* = 1,314): Y (62.8%), N (37.2%);  Religiously affiliated: Yes (*n* = 2,788): Y (63.9%), N (36.1%); No (*n* = 169): Y (53.8%), N (46.2%);  Protestant Denomination: Yes (*n* = 694): Y (64.8%), N (24.2%); Other/No (*n* = 2,094): Y (63.6%), N (36.4%);  R/S: **Religious service attendance**: Some degree but less than once a week (50.3%), once a week or more (36.7%), never (12.9%);  **Importance of religion**: Very (82.9%), Somewhat (29%), Not (2.6%);  **Religious affiliation** | **Chi-squared analyses** for attitudes between R/S variable levels (value NR): **Attitudes toward**:  Taxes on alcoholic beverages: Religiousness = ns: Very important = ns, Somewhat/Not important = ns;  Religious Attendance = ns: 1+ times/week*, <1 times/week = ns;  Religiously affiliated = ns: Yes = ns, No = ns;  Protestant Denomination*: Yes = ns, Other/No = ns;  Minimum legal age for sale of alcohol: Religiousness**: Very important = ns, Somewhat/Not important = ns;  Religious Attendance = ns: 1+ times/week = ns, <1 times/week = ns;  Religiously affiliated = ns: Yes = ns, No = ns;  Protestant Denomination = ns: Yes = ns, Other/No = ns;  Government advertising campaigns: Religiousness = ns: Very important = ns, Somewhat/Not important = ns;  Religious Attendance: 1+ times/week = ns, <1 times/week = ns;  Religiously affiliated = ns: Yes = ns, No = ns;  Protestant Denomination = ns: Yes = ns, Other/No = ns;  School-based prevention programs: Religiousness = ns: Very important = ns, Somewhat/Not important = ns;  Religious Attendance = ns: 1+ times/week = ns, <1 times/week = ns;  Religiously affiliated = ns: Yes = ns, No = ns;  Protestant Denomination = ns: Yes = ns, Other/No = ns;  Alcohol treatment programs: Religiousness = ns: Very important = ns, Somewhat/Not important = ns;  Religious Attendance = ns: 1+ times/week = ns, <1 times/week = ns;  Religiously affiliated = ns: Yes = ns, No = ns;  Protestant Denomination = ns: Yes = ns, Other/No = ns;  Restrictions on hours for alcohol sales: Religiousness**: Very important = ns, Somewhat/Not important = ns;  Religious Attendance = ns: 1+ times/week**, <1 times/week = ns;  Religiously affiliated*: Yes = ns, No = ns;  Protestant Denomination = ns: Yes = ns, Other/No = ns;  More efforts by businesses to stop serving alcohol to drunk customers: Religiousness = ns: Very important = ns, Somewhat/Not important = ns;  Religious Attendance = ns: 1+ times/week**, <1 times/week = ns;  Religiously affiliated = ns: Yes = ns, No = ns;  Protestant Denomination = ns: Yes = ns, Other/No = ns;  Ban sale of alcohol at bakeries, pastry shops, and grocery stores: Religiousness**: Very important = ns, Somewhat/Not important = ns;  Religious Attendance = ns: 1+ times/week**, <1 times/week = ns;  Religiously affiliated = ns: Yes = ns, No = ns;  Protestant Denomination = ns: Yes = ns, Other/No = ns;  Bottles and cans should have more warning messages about alcohol problems than “drink responsibly”: Religiousness = ns: Very important = ns, Somewhat/Not important = ns;  Religious Attendance = ns: 1+ times/week = ns, <1 times/week = ns;  Religiously affiliated = ns: Yes = ns, No = ns;  Protestant Denomination = ns: Yes = ns, Other/No = ns;  Alcohol advertising on television should be banned: Religiousness*: Very important = ns, Somewhat/Not important = ns;  Religious Attendance = ns: 1+ times/week*, <1 times/week = ns;  Religiously affiliated = ns: Yes = ns, No = ns;  Protestant Denomination = ns: Yes = ns, Other/No = ns;  Alcohol manufacturers should be banned from sponsoring sporting/culture events:  Religiousness = ns: Very important = ns, Somewhat/Not important = ns;  Religious Attendance = ns: 1+ times/week***, <1 times/week = ns;  Religiously affiliated = ns: Yes = ns, No = ns;  Protestant Denomination = ns: Yes = ns, Other/No = ns;  Alcohol advertisements should reserve a space for warning messages: Religiousness = ns: Very important = ns, Somewhat/Not important = ns;  Religious Attendance = ns: 1+ times/week = ns, <1 times/week = ns;  Religiously affiliated = ns: Yes = ns, No = ns;  Protestant Denomination = ns: Yes = ns, Other/No = ns;  Alcohol treatment programs should be free and compulsory at health centers, clinics, and general public hospitals: Religiousness = ns: Very important = ns, Somewhat/Not important = ns;  Religious Attendance = ns: 1+ times/week = ns, <1 times/week = ns;  Religiously affiliated = ns: Yes = ns, No = ns;  Protestant Denomination = ns: Yes = ns, Other/No = ns;  It is very easy for persons under 18 to buy alcohol, despite it being illegal:  Religiousness = ns: Very important = ns, Somewhat/Not important = ns;  Religious Attendance = ns: 1+ times/week*, <1 times/week = ns;  Religiously affiliated = ns: Yes = ns, No = ns;  Protestant Denomination = ns: Yes = ns, Other/No = ns;  Increase supervision of businesses that sell alcohol to prevent sale to minors:  Religiousness = ns: Very important = ns, Somewhat/Not important = ns;  Religious Attendance = ns: 1+ times/week = ns, <1 times/week = ns;  Religiously affiliated = ns: Yes = ns, No = ns;  Protestant Denomination = ns: Yes = ns, Other/No = ns;  Others drinking and driving is a threat to my personal safety and the security of my family: Religiousness = ns: Very important = ns, Somewhat/Not important = ns;  Religious Attendance = ns: 1+ times/week = ns, <1 times/week = ns;  Religiously affiliated = ns: Yes = ns, No = ns;  Protestant Denomination = ns: Yes = ns, Other/No = ns;  Most people who drink and drive are alcoholics or problem drinkers: Religiousness = ns: Very important*, Somewhat/Not important = ns;  Religious Attendance = ns: 1+ times/week*, <1 times/week = ns;  Religiously affiliated = ns: Yes = ns, No = ns;  Protestant Denomination = ns: Yes = ns, Other/No = ns;  No problem driving when beginning to feel the effects of alcohol: Religiousness = ns: Very important = ns, Somewhat/Not important = ns;  Religious Attendance = ns: 1+ times/week = ns, <1 times/week = ns;  Religiously affiliated = ns: Yes = ns, No = ns;  Protestant Denomination = ns: Yes = ns, Other/No = ns;  A person drinking and driving will almost certainly be pulled over and detained:  Religiousness = ns: Very important = ns, Somewhat/Not important = ns;  Religious Attendance = ns: 1+ times/week = ns, <1 times/week = ns;  Religiously affiliated = ns: Yes = ns, No = ns;  Protestant Denomination = ns: Yes = ns, Other/No = ns | |
| **Pedersen & Von Soest (2015)**; Norway; survey;  *N* = 458: Convenience sampling of University students during classes;  Christian (NR), Muslim (NR), Other (NR), None (NR)  Alcohol & Cannabis | Attitudes: **Harmfulness of substance** (Morgan, Muetzelfeldt, Muetzelfeldt, Nutt, & Curran, 2010; 1: *Not*; 6: *Very*):  Overall: Alcohol = 4.71 (.75), Cannabis = 4.74 (.97);  Physical: Alcohol = 4.28 (1.09), Cannabis = 4.38 (1.41);  Mental health conditions: Alcohol = 4.36 (1.21), Cannabis = 5 (1.1);  Dependence: Alcohol = 4.50 (1.26), Cannabis = 4.86 (1.29);  Injuries: Alcohol = 5.39 (.83), Cannabis = 4.29 (1.44);  Social consequences: Alcohol = 5.01 (1.02), Cannabis = 5.18 (1.02);  R/S: **Religious affiliation** | | **Multiple linear regression** predicting harmfulness of substances, controlling for gender, site, age, and use of the same substance (*β*, *t*-test):  Religion (Referent = None): Alcohol: Christianity = .01, *t* = .25 (ns); Islam = -.01, *t* = .16 (ns); Other = .04, *t* = .9 (ns);  Cannabis: Christianity = .08, *t* = 1.98* (Christian > None); Islam = .03, *t* = .67 (ns); Other = -.01, *t* = .28 (ns) |
| **Rooney & Gibbons (1966)**; USA; paper questionnaire;  *N* = 353: Interviewers employed door-to-door sampling of individuals in middle-income areas;  women (52.4%), men (47.6%); Aged 18-25 (20.5%), Aged 45+ (31.4%);  Catholic (34%), Protestant (53.5%), Other (12.5%)  Narcotics | Attitudes: **Agreement with social policy change/Liberalization** (1: *Strongly agree*; 4: *Strongly disagree*; Tolerance range = 5-20):  Narcotics laws should be strengthened: Agree (84.5%), Disagree (15%), NR (.6%);  Physicians should be able to treat drug addiction like they treat other illness: Agree (79.9%), Disagree (18.6%), NR (1.4%);  Addict-peddler and non-Addict-peddler should get same punishment: Agree (66.3%), Disagree (26.6%), NR (7.1%);  Physicians and pharmacists who are drug addicts should have license removed and be heavily punished: Agree (61.1%), Disagree (37.1%), NR (17%);  Cannabis should be legalized and purchasable in stores: Agree (14.2%), Disagree (84.7%), NR (1.1%);  General Tolerance (*n* = 317) = 10.6 (NR);  **Belief in misconceptions about users** (1: *Strongly agree*; 4: *Strongly disagree*):  People using drugs, e.g. heroin and cannabis, are usually physical wrecks: Agree (74.2%), Disagree (23.2%), NR = 2.5%);  Cannabis smokers are more likely to commit bad acts, e.g. sex crimes, than non-users: Agree (61.1%), Disagree (36.5%), NR (2.3%);  Almost impossible for drug addict to keep a legitimate job under the influence of narcotics because they cannot stay alert/pay attention: Agree (60.9%), Disagree (36%), NR (3.1%);  Government policies for drug addicts are effective and have resulted in addicts being cured: Agree (36.8%), Disagree (58.9%), NR (4.2%);  Many sex crimes in the US are committed by drug addicts: Agree (30.6%), Disagree (66.3%), NR (3.1%);  More drug addicts are in medical professions compared to general population: Agree (14.5%), Disagree (82.7%), NR (2.8%);  Most addicts are poverty stricken and come from slums: Agree (11.9%), Disagree (85.8%), NR (2.3%);  R/S: **Religious affiliation** (*n* = 312) | | **Mean score differences** in Drug Addiction General Tolerance Score by Religious Affiliation (*n* = 312):  Protestant = 10.5,  Catholic = 10.3,  Other = 12.1  (All seen to be approximately equal) |
| **Russell, Davies, & Hunter (2011)**; UK & USA; Emailed survey link;  *N* = 591: Treatment providers recruited directly or indirectly via Managers of treatment centres and Addiction treatment newsletters;  women (59.4%), men (40.6%); *M*Age (SD) = 45.35 (10.78);  UK (*n* = 372); USA (*n* = 219);  Affiliation (NR);  Alcohol, Illicit drugs, Prescription drugs, Nicotine, Gambling, Sex/pornography, Internet Use, Video games, Shopping, & Food | Attitudes: **Addiction Belief Scale** (Schaler, 1995; 1: *strongly disagree*; 5: *strongly agree*):  Addiction is a disease (range = 10-50): UK = 24.97 (5.77), USA = 31.02 (5.53);  Addiction is a choice (range = 6-30): UK = 17.96 (3.22), USA = 15.24 (3.39);  Addiction is a way of coping with life (range = 2-10): UK = 7.40 (1.35), USA = 6.28 (1.48);  R/S: **Spiritual Belief Scale** (Schaler, 1996): NR | | **Hierarchical Multiple Regressions** for each ABS subscale, including SBS, sex, age, years as an addiction treatment provider, certification, membership of professional group, personal addiction history, and profit status of treatment:  Addiction is a disease: *F*(8, 565) = 26.47***, *R^2^* = 27.3%: SBS (𝛽 = .4***);  Adding country: ∆*F*(1, 564) = 68.55***, ∆*R^2^* = 8%: Country (𝛽 = .32***: USA> UK), SBS (𝛽 = .28***);  Adding country*Profit Status: ∆*F*(1, 563) = 3.84 (ns), *p* = .051, ∆*R^2^* = .4%: Country (𝛽 = .28***: USA> UK), SBS (𝛽 = .27***);  In those with previous addiction (*n* = 199): *F*(8, 184) = 15.54***, *R^2^* = 40.3%: SBS (𝛽 = .48***), Country (𝛽 = .19**, USA> UK);  Adding past treatment attendance, past 12-step attendance, present 12-step attendance, length of 12-step membership, and present abstinence status: ∆*F*(5, 179) = 7.48***, ∆*R^2^* = 12.2%: Country (𝛽 = .14, ns), SBS (𝛽 = .39***);  Addiction is a choice: *F*(8, 565) = 12.89***, *R^2^* = 15.4%: SBS (𝛽 = -.24***);  Adding country: ∆*F*(1, 564) = 48.71***, ∆*R^2^* = 6.7%: Country (𝛽 = -.3***: USA< UK), SBS (𝛽 = -.13**);  Adding country*Profit Status: ∆*F*(1, 563) = 10.80***, ∆*R^2^* = 1.5%: Country (𝛽 = -.22***: USA< UK), SBS (𝛽 = -.12**);  USA-based only (*n* = 219): *F*(7, 208) = 3.74***, *R^2^* = 8.2%: SBS (𝛽 = -.15*);  Adding profit: ∆*F*(1, 207) = 31.42***, ∆*R^2^* = 11.7%: SBS (𝛽 = -.05, ns);  UK-based only (*n* = 372): *F*(7, 350) = 4.07***, *R^2^* = 7.5%: SBS (𝛽 = -.16**);  Adding profit: ∆*F*(1, 349) = 1.74 (ns), ∆*R^2^* = .5%: SBS (𝛽 = .16**);  In those with previous addiction (*n* = 199): *F*(8, 184) = 7.57***, *R^2^* = 24.8%: SBS (𝛽 = -.26**), Country (𝛽 = -.24**, USA< UK);  Adding past treatment attendance, past 12-step attendance, present 12-step attendance, length of 12-step membership, and present abstinence status: ∆*F*(5, 179) = 6.13***, ∆*R^2^* = 11%: Country (𝛽 = -.17*, USA< UK), SBS (𝛽 = -.12, ns);  Addiction as a way of coping: *F*(8, 565) = 9.45***, *R^2^* = 11.8%: SBS (𝛽 = -.14***);  Adding country: ∆*F*(1, 564) = 57.80***, ∆*R^2^* = 8.2%: Country (𝛽 = -.33***: USA< UK), SBS (𝛽 = -.03, ns);  Adding country*Profit Status: ∆*F*(1, 563) = 14.40***, ∆*R^2^* = 2%: Country (𝛽 = -.24***: USA< UK), SBS (𝛽 = -.01, ns);  USA-based only (*n* = 219): *F*(7, 208) = 2.84**, *R^2^* = 8.8%: SBS (𝛽 = -.23***); Adding profit: ∆*F*(1, 207) = 32.56***, ∆*R^2^* = 12.4%: SBS (𝛽 = -.12, ns);  UK-based only (*n* = 372): *F*(7, 350) = 2.32*, *R^2^* = 4.4%: SBS (𝛽 = .07, ns); Adding profit: ∆*F*(1, 349) = 2.92 (ns), ∆*R^2^* = .8%: SBS (𝛽 = .07, ns);  In those with previous addiction (*n* = 199): *F*(8, 184) = 5.29***, *R^2^* = 18.7%: SBS (𝛽 = .02, ns), Country (𝛽 = -.34***, USA< UK);  Adding past treatment attendance, past 12-step attendance, present 12-step attendance, length of 12-step membership, and present abstinence status: ∆*F*(5, 179) = .64 (ns), ∆*R^2^* = 1.8%: Country (𝛽 = -.31***, USA< UK), SBS (𝛽 = .06, ns) |
| **Schaler (1996)**; USA, Canada, Australia; mailed survey;  *N* = 295: Random sample of members of the National Association of Alcoholism and Drug Abuse Counselors (NAADAC), supervisors for Rational Recovery Systems (RRS) groups, addiction treatment providers who are members of the Society of Psychologists in Addictive Behaviors (SPAB);  women (37.1%), men (62.9%); *M*Age (SD) = 44.04 (9.68); Caucasian (94.9%), African-American (2.4%), Hispanic (1%), American Indian (1%), Asian (.3%);  Protestant (27.5%), Catholic (15.6%), Jewish (14.2%), Atheist (7.5%), Agnostic (10.2%), Other (21.7%)  Alcohol | Attitudes: **Addiction Belief Scale** (Schaler, 1995; 1: *strongly disagree*; 5: *strongly agree*; range 18-90 with higher scores related to more belief in the disease model): 54.12 (13.55);  R/S: **Spiritual Belief Scale** (Schaler, 1996): 24.27 (8.55) | | **Stepwise regression** predicting SBS score: ABS: *F*(1,212) = 150.60817***, *R^2^* = .41535 |
| **Schaler (1997)**; USA, Canada, Australia; mailed survey;  *N* = 295: Random sample of members of the National Association of Alcoholism and Drug Abuse Counselors (NAADAC), supervisors for Rational Recovery Systems (RRS) groups, addiction treatment providers who are members of the Society of Psychologists in Addictive Behaviors (SPAB);  women (37.1%), men (62.9%); *M*Age (SD) = 44.04 (9.68); Caucasian (94.9%), African-American (2.4%), Hispanic (1%), American Indian (1%), Asian (.3%);  Protestant (27.5%), Catholic (15.6%), Jewish (14.2%), Atheist (7.5%), Agnostic (10.2%), Other (21.7%)  Alcohol & Drugs | Attitudes: **Addiction Belief Scale** (Schaler, 1995; 1: *strongly disagree*; 5: *strongly agree*; range 18-90 with higher scores related to more belief in the disease model): 54.12 (13.55);  **Beliefs on addiction recovery without treatment** (range = 0-100): 25.07 (24.42)  R/S: **Religious Affiliation**;  **Spiritual Belief Scale** (Schaler, 1996): 24.27 (8.55) | | **Correlation** (*r*) between SBS and ABS: .64***  **Regression** predicting ABS, after controlling for group, gender, drug frequency, marital status, certification, education, abstinence, age, previous addiction, AA attendance, and religious affiliation or SBS:  SBS: 𝛽 = .27***, *R^2^* = .41, *∆R^2^* = .0290;  Religious affiliation (Referent = Atheist): *F* = 2.64 (ns):  Protestant (𝛽 = -.05, *p* = .5114), Catholic (𝛽 = .002, *p* = .9031), Jewish (𝛽 = .1, *p* = .0611), Agnostic (𝛽 = -.03, *p* = .6384) |
| **Sukhwal & Suman (2013)**; India; Questionnaire;  *N* = 236: Students at English-speaking colleges were invited to participate during class;  women (50.8%), men (49.2%); Aged 18-21 (100%);  Affiliation (NR);  Alcohol | Attitudes: **Scale for Assessment of Attitudes toward Drinking and Alcoholism** (Basu, Malhotra, Varma, & Malhotra, 1998):  Men = 83.64 (13.43), Women = 86.53 (11.62);  R/S: **Beliefs and Values Scale** (King et al., 2006): Men = 57.87 (13.66), Women = 57.11 (9.77);  **The Religious Background and Behaviour Questionnaire** (Connors et al., 1996):  God Consciousness: Men = 18.16 (4.99), Women = 18.42 (3.87);  Formal Practices: Men = 13.31 (8.24), Women = 12.28 (8.20) | | **Correlation** between Attitudes and:  Beliefs and Values Scale (*r* = -.19**);  Religious Background and Behaviour Questionnaire (*r* = -.18**):  God consciousness (*r* = -.16*),  Formal practices (*r* = -.16*) |
| **Galanter, Larson, & Rubenstone (1991)**; USA; mailed survey and interview;  *N* = 193: Convenience sampling of members of the Christian Dental and Medical Society (a strong religious, fundamentalist organization);  women (11%), men (89%); *M*Age = 49.1 (12.7)  Assumed Christian (100%);  Alcohol | Attitudes: **Relative effectiveness of Alcoholism treatment for Christian believers** (1: *not at all*; 5: *very much*; range = 1-5):  Psychotropic medication = 1.9 (.8),  Bible and prayer = 3.6 (1.1),  Insight psychotherapy = 2.7 (.9);  **Proportion of patients for whom each treatment would help**: Patients “committed to Christian beliefs”:  AA = 92% (SD = 17.4%),  Psychotherapy = 63.9% (SD = 33.3%),  Prayer by patient = 59.6% (SD = 41.3%);  Patients from “Non-Believing Christian Backgrounds”:  AA = 93.9% (SD = 15%),  Psychotherapy = 63.5% (SD = 33.5%),  Prayer by patient = 19.8% (SD = 34.5%)  R/S: **Gallup poll adapted**: Belief in a personal God who rewards and punishes (78%);  Belief in the devil (98%);  Evangelical Christian beliefs: “Born again” (96%),  Had encouraged someone to accept Jesus as savior (96%),  Every part of Bible is actual word of God to be taken literally (43%) | | **ANOVA** between attitudes towards treatment effectiveness: *F*(2,374) = 182***:  Bible and prayer >Insight psychotherapy> Psychotropic medication  ***t*-test** between proportion of patients for whom a treatment would help for Committed Christian patients vs Patients from non-believing backgrounds:  AA: *t*(188) = -2.2* (Non-believing background> Committed Christian),  Psychotherapy: *t*(188) = .8 (ns),  Prayer by patient: *t*(188) = 13.5*** (Committed Christian > Non-believing background) |
| **Weiss & Moore (1992)**; Israel; questionnaire;  *N* = 533: Teachers who belonged to or worked at a school that sent teachers to an Alcohol and Drug Education training course;  women (58%), men (42%); born in 1930’s (7.7%), born in 1940’s (27.1%), born in 1950’s (45%), born in 1960’s (20.2%);  Jewish (50.7%), Muslim (30.2%), Christian (20.5%);  Alcohol | Attitude: **Agreement with alcohol beliefs** (Caetano, 1989; Moral issue = A+B+C; Disease = D+E):  “Many alcoholics taper off and control their drinking again.” (A):  Jewish: Men (*n* = 41; Agree = 10%, Disagree = 83%), Women (*n* = 229; Agree = 11%, Disagree = 86%);  Muslim: Men (*n* = 129; Agree = 43%, Disagree = 57%), Women (*n* = 32; Agree = 44%, Disagree = 56%);  Christian: Men (*n* = 50; Agree = 50%, Disagree = 50%), Women (*n* = 59; Agree = 58%, Disagree = 42%);  “Most alcoholics drink because they want to.” (B):  Jewish: Men (*n* = 41; Agree = 49%, Disagree = 46%), Women (*n* = 229; Agree = 45%, Disagree = 53%);  Muslim: Men (*n* = 129; Agree = 50%, Disagree = 50%), Women (*n* = 32; Agree = 50%, Disagree = 50%);  Christian: Men (*n* = 50; Agree = 56%, Disagree = 44%), Women (*n* = 59; Agree = 69%, Disagree = 31%);  “Alcoholics are morally weak individuals.” (C):  Jewish: Men (*n* = 41; Agree = 32%, Disagree = 63%), Women (*n* = 229; Agree = 23%, Disagree = 74%);  Muslim: Men (*n* = 129; Agree = 60%, Disagree = 40%), Women (*n* = 32; Agree = 41%, Disagree = 59%);  Christian: Men (*n* = 50; Agree = 42%, Disagree = 58%), Women (*n* = 59; Agree = 42%, Disagree = 58%);  “Alcoholism is an illness.” (D):  Jewish: Men (*n* = 41; Agree = 68%, Disagree = 29%), Women (*n* = 229; Agree = 83%, Disagree = 16%);  Muslim: Men (*n* = 129; Agree = 74%, Disagree = 26%), Women (*n* = 32; Agree = 87%, Disagree = 13%);  Christian: Men (*n* = 50; Agree = 50%, Disagree = 50%), Women (*n* = 59; Agree = 68%, Disagree = 32%);  “To recover, alcoholics have to quit forever” (E):  Jewish: Men (*n* = 41; Agree = 76%, Disagree = 24%), Women (*n* = 229; Agree = 83%, Disagree = 16%);  Muslim: Men (*n* = 129; Agree = 85%, Disagree = 15%), Women (*n* = 32; Agree = 75%, Disagree = 25%);  Christian: Men (*n* = 50; Agree = 60%, Disagree = 40%), Women (*n* = 59; Agree = 64%, Disagree = 36%);  R/S: **Religious affiliation** | | ***F*-test** between religions in agreement with alcohol beliefs:  “Many alcoholics taper off and control their drinking again.”:  Christian ≅ Muslim (*F*(1,524) = 3.35, ns), Jewish < Christian & Muslim (*F*(1,524) = 63.33***);  “Alcoholics are morally weak individuals.”:  Christians ≅ Muslims (*F*(1,525) = 1.69, ns), Jewish < Christian & Muslim (*F*(1,525) = 11.81***);  “Alcoholism is an illness.”:  Jewish ≅ Muslim (*F*(1,531) < 1, ns), Christian < Jewish & Muslim (*F*(1,531) = 17.26***);  “To recover, alcoholics have to quit forever”:  Jewish ≅ Muslim (*F*(1,532) < 1, ns), Christian < Jewish & Muslim (*F*(1,532) = 14.26***);  Moral Issue (A+B+C): *F*(2,517) = 21.29**;  Disease Problem (D+E): *F*(2,529) = 13.6**;  **Percentage** of those that agree:  Belief in Moral Issue (A+B+C) by Religion and Belief Alcoholism is a Disease (D): Inconsistency (no tests run):  Jewish: Agree with D (*n* = 203): Agree with A (11%), Agree with B (43%), Agree with C (27%);  Disagree with D (*n* = 49): Agree with A (18%), Agree with B (53%), Agree with C (22%);  Muslim: Agree with D (*n* = 127): Agree with A (32%), Agree with B (42%), Agree with C (57%);  Disagree with D (*n* = 34): Agree with A (47%), Agree with B (76%), Agree with C (56%);  Christian: Agree with D (*n* = 64): Agree with A (53%), Agree with B (56%), Agree with C (52%);  Disagree with D (*n* = 42): Agree with A (57%), Agree with B (74%), Agree with C (31%); |
| *Note*: If results presented in an article’s table differed from those reported in the text, the data from the table was used; If percentages and number of people conflicted in an article’s reported data, the number of participants was used rather than percentages; ^1^ = A *juramento*, according to the authors, is a religious ritual developed in Mexico in which the user commits to abstinence for a specified time by promising to remain abstinent to a saint, usually The Virgin of Guadalupe; NR = not reported; ns = not significant (*p* > .05); * *p* ≤ .05; ** *p* ≤ .01 ***; *p* ≤ .001. | | | |

References

Anderson, P., & Clement, S. (1987). The AAPPQ Revisited: the measurement of general practitioners’ attitudes to alcohol problems. *British Journal of Addiction*, *82*(7), 753–759. doi: 10.1111/j.1360-0443.1987.tb01542.x

Basu, D., Malhotra, A., Varma, V. K., & Malhotra, R. (1998). Development of a scale to assess attitudes toward drinking and alcoholism. *Indian Journal of Psychiatry*, *40*(2), 158–164.

Best, D. W., Harris, J. C., Gossop, M., Manning, V. C., Man, L. H., Marshall, J., … Strang, J. (2001). Are the Twelve Steps more acceptable to drug users than to drinkers? A comparison of experiences of and attitudes to Alcoholics Anonymous (AA) and Narcotics Anonymous (NA) among 200 substance misusers attending inpatient detoxification. *European Addiction Research*, *7*(2), 69–77. doi: 10.1159/000050719

Bilal, A. M., Makhawi, B., Al-Fayez, G., & Shaltout, A. F. (1990). Attitudes of a sector of the Arab-Muslim population in Kuwait towards alcohol and drug misuse: An objective appraisal. *Drug and Alcohol Dependence*, *26*(1), 55–62. doi: 10.1016/0376-8716(90)90083-Q

Broadus, A. D., & Evans, W. P. (2015). Developing the public attitudes about addiction instrument. *Addiction Research & Theory*, *23*(2), 115–130. doi: 10.3109/16066359.2014.942296

Bugle, L., Jackson, E., Kornegay, K., & Rives, K. (2003). Attitudes of nursing faculty regarding nursing students with a chemical dependency: A national survey. *Journal of Addictions Nursing*, *14*(3), 125–132. doi: 10.1080/10884600390245703

Caetano, R. (1989). Concepts of alcoholism among whites, blacks and hispanics in the United States. *Journal of Studies on Alcohol*, *50*(6), 580–582. doi: 10.15288/jsa.1989.50.580

Christo, G., & Franey, C. (1995). Drug users’ spiritual beliefs, locus of control and the disease concept in relation to Narcotics Anonymous attendance and six-month outcomes. *Drug and Alcohol Dependence*, *38*(1), 51–56. doi: 10.1016/0376-8716(95)01103-6

Chu, D. C., & Sung, H.-E. (2014). Causation of drug abuse and treatment strategy: A comparison of counselors’ perceptions of faith-based and secular drug treatment programs. *International Journal of Offender Therapy and Comparative Criminology*, *58*(4), 496–515. doi: 10.1177/0306624X12462855

Connors, G. J., Tonigan, J. S., & Miller, W. R. (1996). A measure of religious background and behavior for use in behavior change research. *Psychology of Addictive Behaviors*, *10*(2), 90–96. doi: 10.1037/0893-164X.10.2.90

Crothers, C. E., & Dorrian, J. (2011). Determinants of nurses’ attitudes toward the care of patients with alcohol problems. *International Scholarly Research Notices: Nursing*, *2011*, 1–11. doi: 10.5402/2011/821514

Cuadrado, M. (2014). Hispanic use of juramentos and Roman Catholic Priests as auxiliaries to abstaining from alcohol use/misuse. *Mental Health, Religion and Culture*, *17*(10), 1015–1022. doi: 10.1080/13674676.2014.995074

Cuadrado, M., & Lieberman, L. (2011). The Virgin of Guadalupe as an ancillary modality for treating Hispanic substance abusers: Juramentos in the United States. *Journal of Religion and Health*, *50*(4), 922–930. doi: 10.1007/s10943-009-9304-4

Day, E., Gaston, R. L., Furlong, E., Murali, V., & Copello, A. (2005). United Kingdom substance misuse treatment workers’ attitudes toward 12-step self-help groups. *Journal of Substance Abuse Treatment*, *29*(4), 321–327. doi: 10.1016/j.jsat.2005.08.009

Dermatis, H., Guschwan, M. T., Galanter, M., & Bunt, G. (2004). Orientation toward spirituality and self-help approaches in the therapeutic community. *Journal of Addictive Diseases*, *23*(1), 39–54. doi: 10.1300/J069v23n01_04

Dermatis, H., James, T., Galanter, M., & Bunt, G. (2010). An exploratory study of spiritual orientation and adaptation to therapeutic community treatment. *Journal of Addictive Diseases*, *29*(3), 306–313. doi: 10.1080/10550887.2010.489443

Diallo, A. (2013). Clients’ willingness to incorporate religion or spirituality in counseling: A brief report. *Rehabilitation Counseling Bulletin*, *56*(2), 120–122. doi: 10.1177/0034355212439425

Droubay, B. A., & Butters, R. P. (2020). Pornography, religiosity, and social work. *Journal of Social Work*, *20*(5), 557–575. doi: 10.1177/1468017319852599

Edelstein, O. E., Wacht, O., Grinstein-Cohen, O., Reznik, A., Pruginin, I., & Isralowitz, R. (2020). Does religiosity matter? University student attitudes and beliefs toward medical cannabis. *Complementary Therapies in Medicine*, *51*(102407). doi: 10.1016/j.ctim.2020.102407

Feagin, J. R. (1964). Prejudice and relegious types: A focused study of southern fundamentalists. *Journal for the Scientific Study of Religion*, *4*(1), 3. doi: 10.2307/1385200

Flórez, K. R., Derose, K. P., Breslau, J., Griffin, B. A., Haas, A. C., Kanouse, D. E., … Williams, M. V. (2015). Acculturation and drug use stigma among Latinos and African Americans: An examination of a church-based sample. *Journal of Immigrant and Minority Health*, *17*(6), 1607–1614. doi: 10.1007/s10903-015-0161-9

Galanter, M., Larson, D., & Rubenstone, E. (1991). Christian psychiatry: The impact of evangelical belief on clinical practice. *The American Journal of Psychiatry*, *148*(1), 90–95. doi: 10.1176/ajp.148.1.90

Globetti, G., Alsikafi, M., & Christy, E. G. (1977). Permissive attitudes toward alcohol abuse among young military dependents. *Journal of Drug Education*, *7*(2), 99–107. doi: 10.2190/V1MW-R0XM-9DPG-75P4

Goldfarb, L. M., Galanter, M., McDowell, D., Lifshutz, H., & Dermatis, H. (1996). Medical student and patient attitudes toward religion and spirituality in the recovery process. *The American Journal of Drug and Alcohol Abuse*, *22*(4), 549–561. doi: 10.3109/00952999609001680

Gritsenko, V., Konstantinov, V., Reznik, A., & Isralowitz, R. (2020). Russian Federation medical student knowledge, attitudes and beliefs toward medical cannabis. *Complementary Therapies in Medicine*, *48*(102274). doi: 10.1016/j.ctim.2019.102274

Gurel, M., & Spain, M. D. (1977). Differences in attitudes toward alcoholism in graduates of two schools of nursing. *Psychological Reports*, *41*, 1285–1286. doi: 10.2466/pr0.1977.41.3f.1285

Hatchett, B. (2002). *Attitudes of older African American women about alcohol abuse*. Lewiston, NY: Edwin Mellen Pr.

Hatchett, B., Holmes, K. Y., Bryan-Young, R., & Patterson, B. (2011). Attitudes held by members of the clergy about alcohol. *The Journal of Pastoral Care & Counseling*, *65*(1–2), 1–13. doi: 10.1177/154230501106500104

Hecker, L. L., Trepper, T. S., Wetchler, J. L., & Fontaine, K. L. (1995). The influence of therapist values, religiosity and gender in the initial assessment of sexual addiction by family therapists. *The American Journal of Family Therapy*, Vol. 23, pp. 261–272.

Hendrix, M. J., Sabritt, D., McDaniel, A., & Field, B. (1987). Perceptions and attitudes toward nursing impairment. *Research in Nursing & Health*, *10*(5), 323–333. doi: 10.1002/nur.4770100506

Hshieh, S. Y., & Srebalus, D. J. (1997). Alcohol treatment issues: Professional differences. *Alcoholism Treatment Quarterly*, *15*(4), 63–73. doi: 10.1300/J020V15N04_05

Jolly, S., & Orford, J. (1983). Religious observance, attitudes towards drinking, and knowledge about drinking, amongst university students. *Alcohol and Alcoholism*, *18*(3), 271–278. doi: 10.1093/oxfordjournals.alcalc.a044373

King, M., Jones, L., Barnes, K., Low, J., Walker, C., Wilkinson, S., … Tookman, A. (2006). Measuring spiritual belief: Development and standardization of a beliefs and values scale. *Psychological Medicine*, *36*(3), 417–425. doi: 10.1017/S003329170500629X

Lawrence, R. E., Rasinski, K. A., Yoon, J. D., Koenig, H. G., Meador, K. G., & Curlin, F. A. (2012). Physicians’ beliefs about faith-based treatments for alcoholism. *Psychiatric Services*, *63*(6), 597–604. doi: 10.1176/appi.ps.201100315

Linsky, A. S. (1965). Religious differences in lay attitudes and knowledge on alcoholism and its treatment. *Journal for the Scientific Study of Religion*, *5*, 41–50. doi: 10.2307/1384253

Linsky, A. S., Fagan, R. J., & Heinemann, E. (1964). Attitudes on alcoholism in a tuberculosis hospital. *Washington State Health Department*.

Loewenthal, K. M., MacLeod, A. K., & Cinnirella, M. (2002). Are women more religious than men? Gender differences in religious activity among different religious groups in the UK. *Personality and Individual Differences*, *32*(1), 133–139. doi: 10.1016/S0191-8869(01)00011-3

Loewenthal, K. M., MacLeod, A. K., Cook, S., Lee, M., & Goldblatt, V. (2003). Beliefs about alcohol among UK Jews and Protestants: Do they fit the alcohol-depression hypothesis? *Social Psychiatry and Psychiatric Epidemiology: The International Journal for Research in Social and Genetic Epidemiology and Mental Health Services*, *38*(3), 122–127. doi: 10.1007/s00127-003-0609-4

Lucchetti, G., Koenig, H. G., Pinsky, I., Laranjeira, R., & Vallada, H. (2014). Religious beliefs and alcohol control policies: A Brazilian nationwide study. *Revista Brasileira de Psiquiatria*, *36*(1), 4–10. doi: 10.1590/1516-4446-2012-1051

Marcus, A. M. (1980). *The alcoholism questionnaire: Administration, scoring and interpretation*. Toronto, Canada: Alcohol and Drug Addiction Research Association.

Martin, C. S., Earleywine, M., Musty, R. E., Perrine, M. W., & Swift, R. M. (1993). Development and validation of the Biphasic Alcohol Effects Scale. *Alcoholism: Clinical and Experimental Research*, *17*(1), 140–146. doi: 10.1111/j.1530-0277.1993.tb00739.x

Metrikin, A. S., Galanter, M., Dermatis, H., & Bunt, G. (2003). Somatization, anxiety and depression in a drug-free residential therapeutic community. *The American Journal on Addictions*, *12*(1), 60–70. doi: 10.1080/10550490390143367

Miller, W. R., & Marlatt, G. A. (1984). *Manual for the Comprehensive Drinker Profile*. Odessa, FL: Psychological Assessment Resources.

Morgan, C. J. A., Muetzelfeldt, L., Muetzelfeldt, M., Nutt, D. J., & Curran, H. V. (2010). Harms associated with psychoactive substances: Findings of the UK national drug survey. *Journal of Psychopharmacology*, *24*(2), 147–153. doi: 10.1177/0269881109106915

O’Connor, J. (1978). The young drinkers: A cross-national study of social and cultural influences. In *Tavistock Publications*. London: Tavistock Publications.

Pedersen, W., & Von Soest, T. (2015). Which substance is most dangerous? Perceived harm ratings among students in urban and rural Norway. *Scandinavian Journal of Public Health*, *43*(4), 385–392. doi: 10.1177/1403494815576267

Pennington, D. F., & Passey, G. E. (1960). *Development of a scale for the assessment of knowledge concerning alcohol and its use*. Montgomery, AL: Alabama Commission on Alcoholism.

Plante, T. G., & Boccaccini, M. T. (1997). The Santa Clara strength of religious faith questionnaire. *Pastoral Psychology*, *45*(5), 375–387. doi: 10.1007/BF02230993

Ritsher, J. B., Otilingam, P. G., & Grajales, M. (2003). Internalized stigma of mental illness: Psychometric properties of a new measure. *Psychiatry Research*, *121*(1), 31–49. doi: 10.1016/j.psychres.2003.08.008

Ronzani, T. M., Higgins-Biddle, J., & Furtado, E. F. (2009). Stigmatization of alcohol and other drug users by primary care providers in Southeast Brazil. *Social Science and Medicine*, *69*(7), 1080–1084. doi: 10.1016/j.socscimed.2009.07.026

Rooney, E. A., & Gibbons, D. O. N. C. (1966). Social reactions to “Crimes without victims.” *Social Problems*, *13*(4), 401–410. doi: 10.1525/sp.1966.13.4.03a00040

Russell, C., JB, D., SC, H., Russell, C., Davies, J. B., & Hunter, S. C. (2011). Predictors of addiction treatment providers’ beliefs in the disease and choice models of addiction. *Journal of Substance Abuse Treatment*, *40*(2), 150–164. doi: 10.1016/j.jsat.2010.09.006

Santos da Silveira, P., Casela, A. L. M., Monteiro, É. P., Lubambo Ferreira, G. C., Tibúrcio de Freitas, J. V., Tibúrcio de Freitas, J. V., … Ronzani, T. M. (2018). Psychosocial understanding of self-stigma among people who seek treatment for drug addiction. *Stigma and Health*, *3*(1), 42–52. doi: 10.1037/sah0000069

Schaler, J. A. (1995). The addiction belief scale. *Substance Use and Misuse*, *30*(2), 117–134. doi: 10.3109/10826089509060737

Schaler, J. A. (1996). Spiritual thinking in addiction-treatment providers: The spiritual belief scale (SBS). *Alcoholism Treatment Quarterly*, *14*(3), 7–33. doi: 10.1300/J020V14N03_03

Schaler, J. A. (1997). Addiction beliefs of treatment providers: Factors explaining variance. *Addiction Research and Theory*, *4*(4), 367–384. doi: 10.3109/16066359709002970

Seaman, J., & Mannello, T. (1978). *The Seaman-Mannello Scale*. Arlington, VA: National Institute on Alcohol Abuse and Alcoholism.

Strassburger, F., & Strassburger, Z. (1965). Measurement of attitudes toward alcohol and their relation to personality variables. *Journal of Consulting Psychology*, *29*(5), 440–445. doi: 10.1037/h0022487

Sukhwal, M., & Suman, L. N. (2013). Spirituality, religiosity and alcohol related beliefs among college students. *Asian Journal of Psychiatry*, *6*(1), 66–70. doi: 10.1016/j.ajp.2012.08.012

Weiss, S., & Moore, M. (1992). Perception of alcoholism among Jewish, Moslem and Christian teachers in Israel. *Journal of Drug Education*, *22*(3), 253–260. doi: 10.2190/50PQ-LN7K-RFVJ-F61N
